# CRISPR/Cas9-based genome-wide screening of *Dictyostelium*

**DOI:** 10.1038/s41598-022-15500-3

**Published:** 2022-07-02

**Authors:** Takanori Ogasawara, Jun Watanabe, Remi Adachi, Yusuke Ono, Yoichiro Kamimura, Tetsuya Muramoto

**Affiliations:** 1 Department of Biology, Faculty of Science, Toho University, 2-2-1 Miyama, Funabashi, Chiba 274-8510 Japan; 2 Laboratory for Cell Signaling Dynamics, RIKEN, Center for Biosystems Dynamics Research (BDR), Suita, Osaka 565-0874 Japan

**Keywords:** Dictyostelium discoideum, CRISPR-Cas9 genome editing, High-throughput screening

## Abstract

Genome-wide screening is powerful method used to identify genes and pathways associated with a phenotype of interest. The simple eukaryote *Dictyostelium discoideum* has a unique life cycle and is often used as a crucial research model for a wide range of biological processes and rare metabolites. To address the inadequacies of conventional genetic screening approaches, we developed a highly efficient CRISPR/Cas9-based genome-wide screening system for *Dictyostelium*. A genome-wide library of 27,405 gRNAs and a kinase library of 4,582 gRNAs were compiled and mutant pools were generated. The resulting mutants were screened for defects in cell growth and more than 10 candidate genes were identified. Six of these were validated and five recreated mutants presented with growth abnormalities. Finally, the genes implicated in developmental defects were screened to identify the unknown genes associated with a phenotype of interest. These findings demonstrate the potential of the CRISPR/Cas9 system as an efficient genome-wide screening method.

## Introduction

Simple eukaryotes are powerful tools that are widely used to identify novel components of cellular processes and are known to be useful in genetic screening. The social amoeba *Dictyostelium discoideum* is a simple and valuable biomedical model that is recognised by the National Institute of Health for studying the functions of proteins associated with human diseases^1–3^. Moreover, because of its unique developmental life cycle and capacity to produce specific secondary metabolites and signalling molecules, *Dictyostelium* can also be used to study multicellular features, such as cell–cell communication and cell migration, and the production of chemical compounds and substances, such as aromatic polyketides^4–8^. In the early 1980s, various morphological mutants were isolated through genetic screening using chemical mutagens and ultraviolet irradiation. However, because of the limitations of linkage analysis based on parasexual genetics, it was difficult to go from the phenotype to the genotype^9–11^. During the 1990s, restriction enzyme-mediated integration (REMI) was a popular method to isolate and analyse mutants causing morphological defects^12^. A drug-resistance gene inserted into the genome can be easily recovered along with the flanking DNA sequences, making it possible to efficiently go from the phenotype to the genotype^13,14^. Hence, REMI is a forward genetic approach used to identify genes responsible for the novel components of various biological processes, such as cell migration, chemotaxis and development^15–19^. Although other techniques for genetic screening, including antisense RNA, RNA interference (RNAi) and CRISPR interference (CRISPRi), have been developed to identify partial loss-of-function mutations^20–23^, none of these techniques are widely used in the genetic screening of *Dictyostelium*.

Improvements in sequencing technology have led to the development of novel approaches for the genetic screening of *Dictyostelium*. For example, mutagenesis using the chemical mutagen *N*-methyl-*N*′-nitro-*N*-nitrosoguanidine (NTG) followed by whole-genome sequencing enables the direct identification of mutated genes^24^. Most NTG-induced single-nucleotide variants are G > A transitions that result in the random introduction of new stop codons and missense mutations throughout the genome, leading to loss-of-function, partial loss-of-function or gain-of-function mutants. If several independent mutations lead to nucleotide variants in the same gene, it is likely that the gene is responsible for the phenotype. REMI-seq enables the high-throughput and quantitative identification of REMI-mutant insertion sites^25^. Resources for the genome-wide loss-of-function mutants generated by REMI-seq technology are distributed by stock centres and are widely used by the research community studying *Dictyostelium*. The major considerations to genetic screening are saturation of mutation in the mutant pool and ease of identifying mutation sites. It has been indicated that whole-genome sequencing of multiple strains is not cost effective approach and that loss-of-function mutants identified via REMI rarely result in gain-of-function mutations in genes that suppress the original phenotype^26–28^. Thus, the use of a variety of approaches is advantageous for the genetic screening of *Dictyostelium*.

Our group developed CRISPR/Cas9-based approaches to study *Dictyostelium*. The transient expression of Cas9 from *Streptococcus pyogenes* (SpCas9) and guide RNA (gRNA) of the target gene resulted in the generation with mutants of knockouts, knock-ins and precise nucleotide substitution with high efficiency (> 50%)^23,29–31^. Mutants can also be generated for genetic screening through gene targeting via homologous recombination^32,33^. However, such methods that use a gene-by-gene approach are not suitable for genome-wide screening. Creating genome-wide loss-of-function libraries using CRISPR/Cas9 gene-editing technology is an alternative method for screening of human cells^34,35^. Such methods have been applied in studies of various biological processes, including drug resistance and sensitivity, oncogenesis, repair of DNA double-strand breaks and the formation of gastric organoids^35–37^. However, to the best of our knowledge, no studies have used CRISPR/Cas9 technology for genome-wide screening of *Dictyostelium*.

Among all mutagenesis techniques currently in use, CRISPR screening has several advantages. First, it is associated with more precise gene targeting with fewer off-target effects compared with traditional technologies such as RNAi. Although complete exclusion is not possible, the off-target effects can be significantly reduced by choosing unique target sequences because DNA targeting specificity is dependent on the locations of the mismatches between gRNA and target DNA^38,39^. Second, the effects of permanent loss-of-function rather than transient knockdown can be observed. Thus, long-term phenotypic assays of more than 1 week are possible. Third, gRNA is a short RNA that can be synthesised easily and in a cost effective manner, thus allowing genome-wide genes to be targeted for analysis. Fourth, the use of CRISPR libraries does not require special equipment for automation and high-throughput screening. Despite these advantages, screening for *Dictyostelium* using CRISPR transient expression vectors has some limitations, such as the potential loss of expression vectors through repeated cell division. To overcome this limitation, CRISPR libraries were established that allow the stable expression of Cas9 and gRNAs to generate mutant pools to identify the genes responsible for the phenotype of interest. Additionally, two sets of gRNA libraries of all kinase and genome-wide genes were designed to validate the mutants obtained through genetic screening. Our findings demonstrate the utility of the proposed method for forward genetic screening of *Dictyostelium*.

## Results

### CRISPR/Cas9 stable expression vector can be used to generate mutant pools and minimise off-target effects

**Figure 1:**
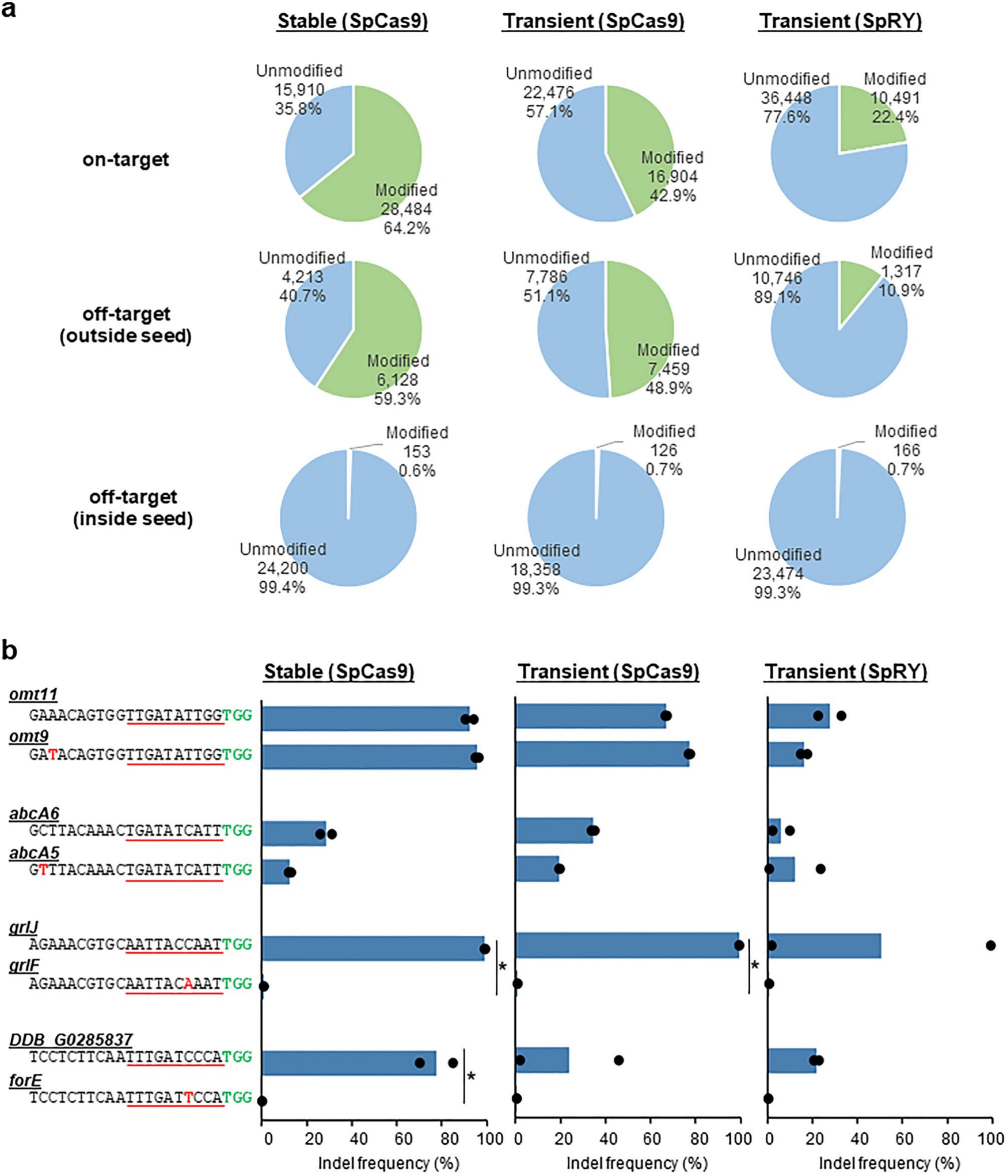
Detection of off-target mutations induced by CRISPR/Cas9 stable and transient expression vectors. (**a**) Percentage of on-target and off-target via three CRISPR vectors. The numbers and percentages of modified and unmodified loci. Outside seed and inside seed represent the presence of mismatches outside and inside the seed region of the target sequence, respectively. (**b**) Indel frequency for each on-target and off-target sequence. Mismatch sequences within off-targets and PAM sequences are presented in red and green letters, respectively. Seed sequence is underlined in red. Data represent two biological repeats and individual data points are shown. *p*-values were calculated using a one-way Welch two-sample *t*-test (*p < 0.05).

All-in-one CRISPR/Cas9 transient expression vectors have been extensively used to generate a wide range of genomic mutations in *Dictyostelium*^23,31^. However, vectors lacking nuclear plasmid sequences are degraded through repeated cell division and, are thus not suitable for genetic screening that requires several weeks for a phenotypic assay. Therefore, the extrachromosomal-based all-in-one CRISPR/Cas9 stable expression vectors, pTM1369 and pTM1709 were developed (Supplementary Fig. 1). Because the long-term expression of SpCas9 and gRNA leads to nonspecific and unintended mutations at off-target sites, the efficiency of vector-mediated editing at the on- and off-target loci was evaluated. The efficiency of conventional methods using transient expression vectors to edit four target genes (*omt11*, *abcA6*, *grlJ*, and *DDB_G0285837*) was only ~ 50%, whereas that of stable expression vectors was higher at ~ 70%, with less variability over repeated transformations (Fig. 1). At off-target sites containing one mismatched nucleotide (*omt9*, *abcA5*, *grlF* and *forE*), the efficiency of editing varied with the position of the mismatch. Mismatches within the seed sequence (< 10 nucleotides at the 3′ end of gRNA; *grlF* and *forE*) reduced editing activity to a greater extent (~ 0.5%) than mismatches outside the seed sequence (*omt9* and *abcA5*) (~ 50%) (Fig. 1). These results are consistent with those of previous studies which indicate that the seed sequence is involved in target recognition and that mismatches at inside of the seed sequence are less tolerated than outside the region^39,40^. Therefore, if highly specific gRNAs are selected using conventional web tools, such as Cas-OFFinder^41^, the off-target effect via stable expression vectors is almost negligible or comparable to that of transient expression vectors.

The Cas9 variant SpRY can recognise the non-canonical protospacer adjacent motif (PAM) flanking the target site and exhibits robust activities over a wide range of *Dictyostelium* target sites^29^. Analysing the editing efficiency via transient expression revealed that the efficiency of SpRY was approximately half of that of on-target SpCas9. In contrast, the off-target regions with a mismatch of one base in the seed sequence resulted in almost the same rate of unintended editing, suggesting that SpRY was useful for target expansion but not as highly efficient as SpCas9.

### Construction of gRNA libraries targeting genome-wide and kinase genes

**Figure 2:**
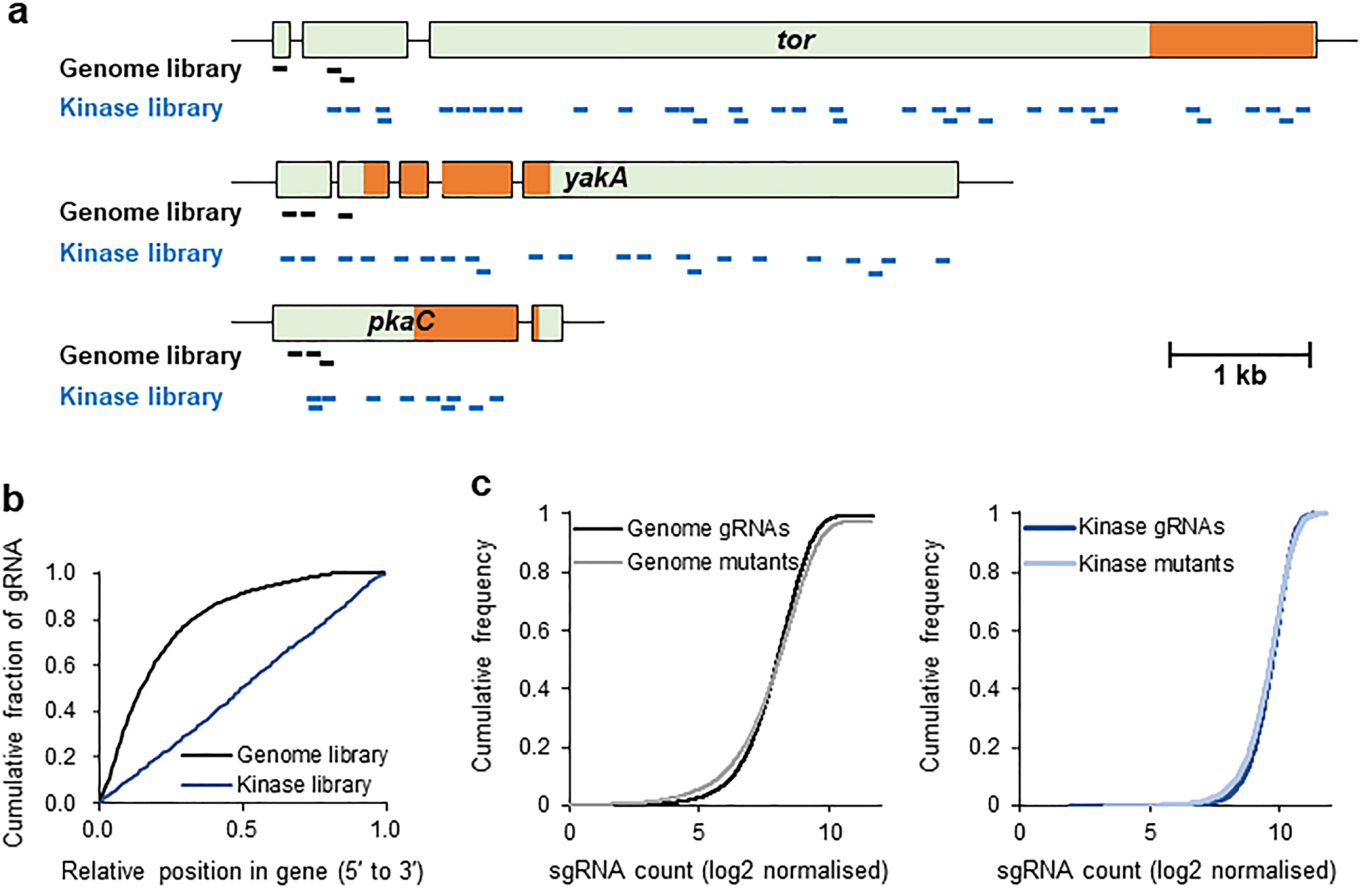
Generation of a genome-wide and kinase sgRNA plasmid library. (**a**) Target gRNA positions in the genome-wide and kinase libraries. Target sequences in genome library were designed within 400 bp of the start codon, while kinase libraries were designed with five gRNAs per 1 kb of each gene. Three representative genes are shown. The kinase domain of each gene is indicated by an orange box. (**b**) Distribution of designed gRNAs in genes. (**c**) Deep-sequencing analysis of the gRNAs in plasmid libraries and genome-wide (left) and kinase (right) mutant pools.

Up to three gRNAs were designed for each protein-coding gene in the range of + 1 to + 400 bp relative to the start codon of the *Dictyostelium* genome and 27,405 gRNAs were identified, covering 84.7% of genes (total 10,269 genes), with at least one gRNA per gene (Fig. 2a and Supplementary Data 1). These gRNAs were cloned into the vector pTM1369 stably expressing Cas9 and single guide RNA (sgRNA) to generate a genome-wide sgRNA plasmid pool. Since the target sequences were designed from the start codon side of the gene, the indels (e.g., insertions, deletions and point mutations) were more likely to contain mutations that significantly disrupt gene function compared with the targets at the end of the gene, thus rendering the sequences suitable for screening the genes responsible for the phenotype of interest. To increase the number of potential target genes, up to five gRNAs were designed for each protein-coding gene over the entire region using different computational programmes, which finally identified 53,397 gRNAs covering 92.7% of 11,238 genes (Supplementary Data 2). Non-coding RNAs, tRNAs, pseudogenes and extrachromosomal genes were excluded from the whole-gene list. Genes for which the designed gRNAs did not meet the criteria were characterised by high sequence homology between genes, including retro-transposable and transposable elements, short genes of a few hundred base pairs, and those with a GC content of < 30%. Moreover, gRNAs were designed for all 283 kinases, and 5 gRNAs per 1 kb were selected (Fig. 2a). The number of selected gRNAs varied with the length of each gene. In total, 4,582 gRNAs were selected (average, 16.1; range, 3–69) (Supplementary Data 3) and cloned into the CRISPR/Cas9 stable expression vector pTM1709 to generate a kinase sgRNA plasmid pool. Genome editing introduced indels into the genome around the gRNAs; therefore, tiling the screens with five gRNAs per 1 kb can provide a precise understanding of the underlying functional domains and protein interaction sites within a gene through a wide variety of genomic mutations. Indeed, the genome-wide sgRNA plasmid had no targets in the kinase domains of *tor*, *yakA* and *pkaC*, whereas the kinase sgRNA plasmid pool was designed with multiple targets within the domain. These differences in design positions were also observed in the distribution of the gRNAs within a gene (Fig. 2b).

To evaluate the quality of the libraries, the genome-wide and kinase plasmid libraries were sequenced at a depth of 193 × (5,278,088 reads) and 226 × (1,033,731 reads), which revealed 27,232 (99.4%) and 4,582 (100%) gRNAs, respectively (Supplementary Fig. 2). A small number of gRNAs were under- or over-represented in both libraries, while 85% and 98% of gRNAs fell within tenfold differences in frequency, respectively (Fig. 2c). Next, mutant pools were generated through eight independent transformations of the genome-wide and kinase sgRNA plasmid libraries. A single electroporation generated approximately 5 × 10^5^ transformants, thus allowing the preparation of pooled mutants derived from 4 × 10^6^ clones. Genomic DNA (gDNA) isolated from the mutant pools were deep-sequenced at a depth of 505 × (13,841,784 reads) and 3,393 × (15,544,559 reads), revealing that 26,708 (99.5%) and 4,582 (100%) of the gRNAs were present in the mutant pools, respectively (Fig. 2c). Seven gRNAs detected in the mutant pool were not present in the genome-wide sgRNA plasmid pool, possibly due to differences in sequencing depth. Compared with the genome-wide sgRNA plasmid library, numerous gRNAs were under-represented in the mutant pool, possibly because the gRNAs targeted essential genes. Gene ontology (GO) analysis of reduced genes revealed that the terms associated with essential biological processes, such as translation and membrane function, were significantly over-represented (Supplementary Table 1). These results also confirmed that the new CRISPR vectors and designed gRNAs are functional in CRISPR-based mutagenesis.

### Identification of candidate kinases regulating cell growth

**Figure 3:**
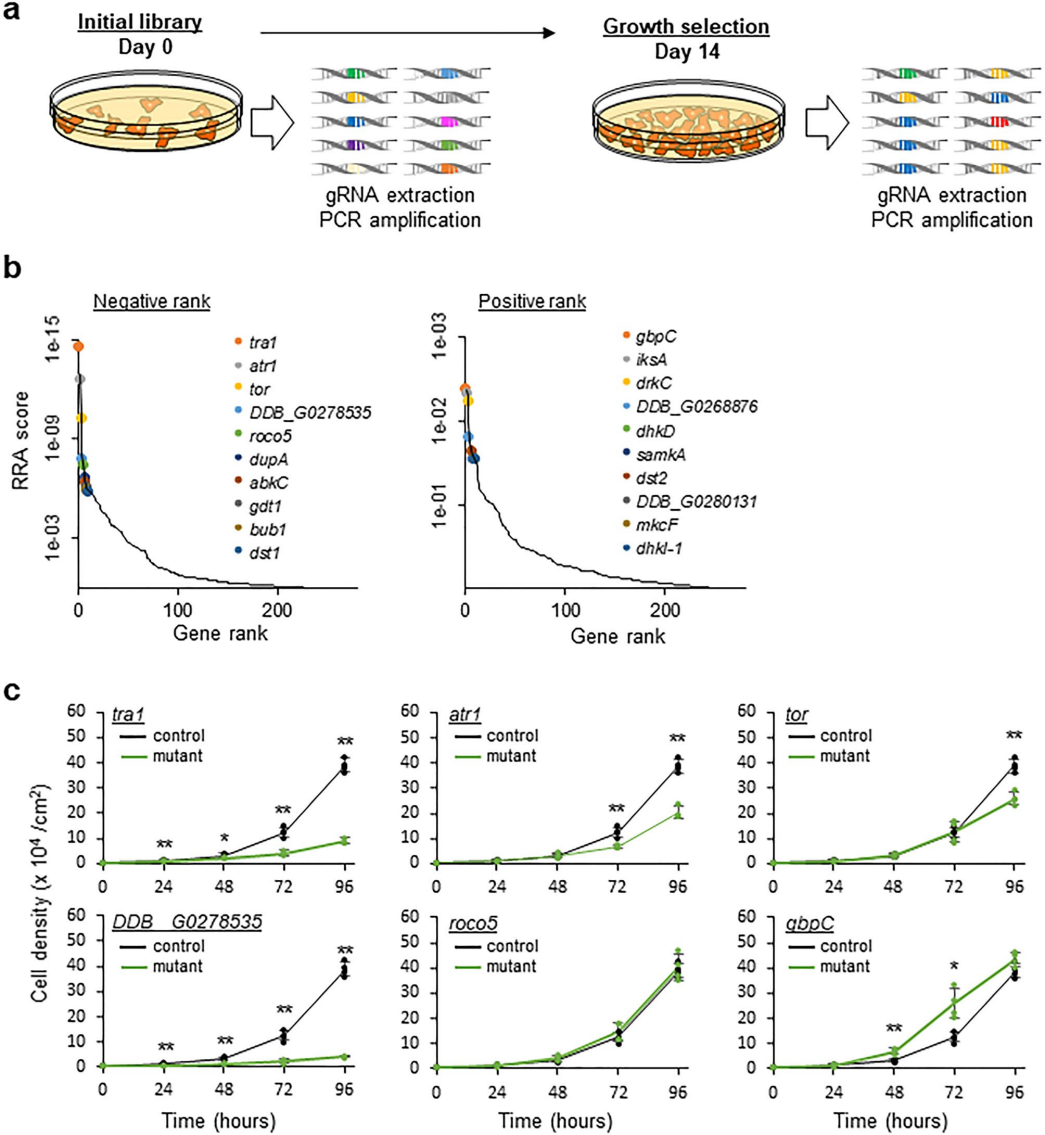
Genetic screening using the kinase sgRNA library and genetic validation of candidate genes regulating cell growth. (**a**) Schematic overview of genetic screening for cell growth with the kinase gRNA library. (**b**) Candidate genes identified by CRISPR-based screening. Data analysis using MAGeCK revealed under- (left) and over-represented (right) gRNAs and ranked the corresponding genes with RRA scores. Low RRA scores indicate stronger enrichment. (**c**) Growth curves of recreated CRISPR mutants. Data points represent the mean ± SD of 3–4 biological repeats and individual data points are shown. Samples were compared with Welch *t*-test; *p < 0.05; **p < 0.01.

Since the generated mutant pools contained almost all of the designed gRNAs, the possibility of isolating the desired phenotype and identifying the gene responsible for it was examined. We particularly focussed on the effects on growth of mutations in specific protein kinase genes; therefore, three independent mutant pools expressing kinase sgRNAs were generated. A fraction of the transformants was used as control (Day 0), while the remainder was cultured for an additional 14 days (~ 37 generations). gDNA was extracted from Day 0 and 14, the sgRNA region was amplified by PCR and the distribution of the gRNAs was determined via deep-sequencing (Fig. 3a & Supplementary Fig. 2). Allocating scores to the changes in the number of the gRNAs revealed that many were under- or over-represented after 14 days of cell proliferation. The MAGeCK (Model-based Analysis of Genome-wide CRISPR/Cas9 Knockout) algorithm (http://liulab.dfci.harvard.edu/Mageck) uses Robust Rank Aggregation (RRA) scores for identifying robust hits from CRISPR screens of the top 10 under- and over-represented genes^42^. The false discovery rate of the top 10 under-represented genes was < 5.9 × 10^−4^ as calculated by MAGeCK, whereas even the most over-represented gene, *gbpC*, had a non-significance score of 0.59 (Fig. 3b & Supplementary Table 2). The top 10 under-represented genes were classified into GO terms related to the cell-cycle checkpoint and cell-cycle regulation (Supplementary Table 3), indicating that pooled CRISPR screening can highlight the relevant gene sets responsible for the desired phenotype.

To determine whether the identified mutants were due to growth defects caused by gene disruption rather than an artefact of genetic screening, five under-represented and one over-represented genes were selected and CRISPR-mediated mutants were recreated using gRNA identified by the screening. Mutants were obtained from all six genes; frameshift mutations were observed in four genes, whereas only non-frameshifted in-frame indel mutants (e.g., single amino acid deletion) were isolated from the other two (Supplementary Fig. 3). One possible explanation for this could be that frameshift mutants could not be obtained because the gene was essential. Comparing the growth rates with the parental strain AX2 indicated that the mutants of *tra1*, *atr1*, *tor* and *DDB_G0278535* reduced the proliferation rates (Fig. 3c), whereas knockout of *gbpC* as a representative over-represented gene, accelerated the growth rate. In summary, the growth phenotype of the mutants was validated, and the candidate genes involved in fundamental biological processes were identified via CRISPR screening.

### Screening of the genome-wide and kinase gRNA libraries to identify genes regulating development

**Figure 4:**
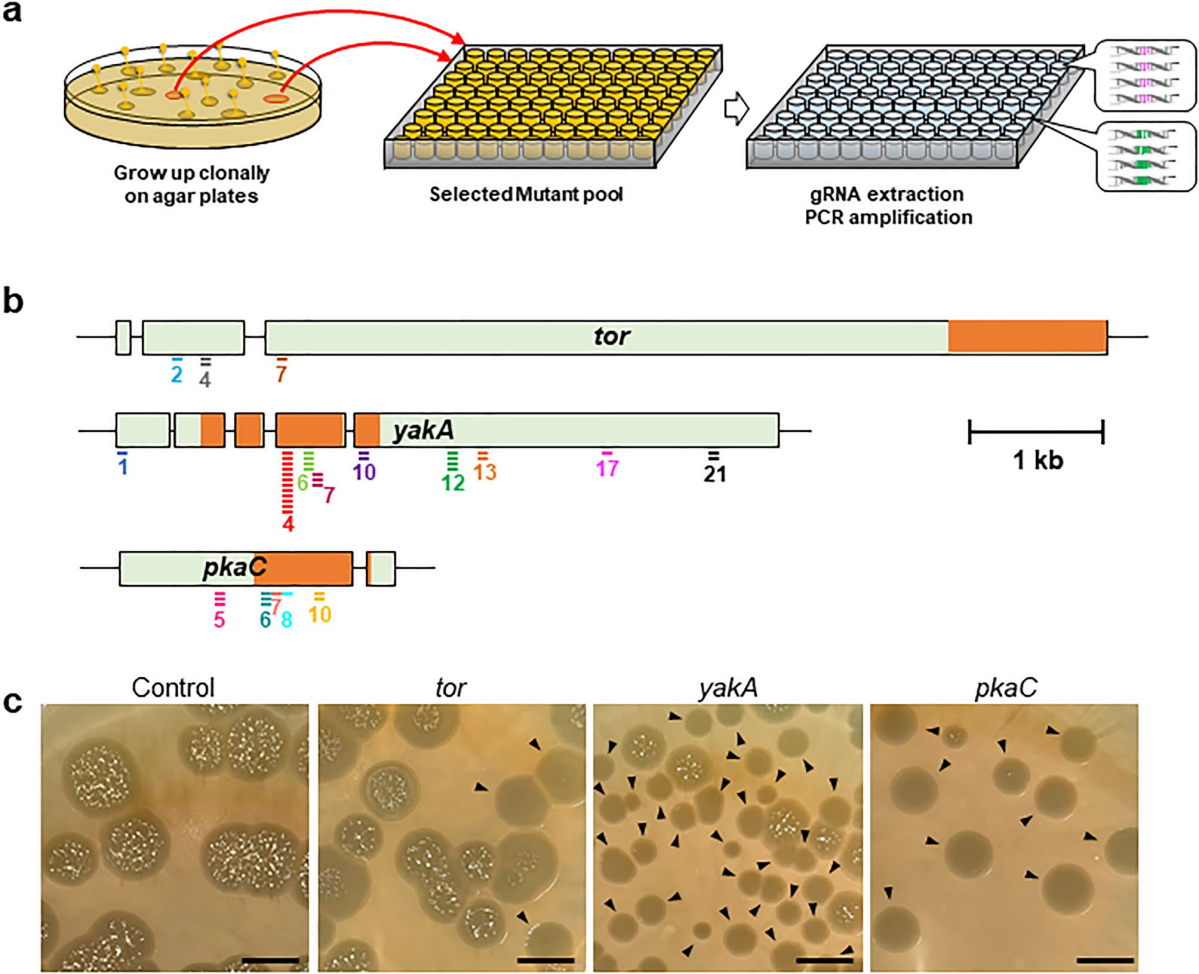
Genetic screening using the CRISPR sgRNA libraries and genetic validation of candidate genes regulating development. (**a**) Schematic overview of genetic screening to identify developmental defective mutants. Mutant pools expressing genome-wide and kinase sgRNA libraries were inoculated on nutrient agar plates with bacteria as a food source and allowed to grow and develop. Isolated plaques exhibiting developmental defects, including aggregation-less and abnormal fruiting bodies, were transferred to 96-well plates. (**b**) Distribution of target sequences detected in developmental mutants. The numbers indicate the serial numbers of gRNAs for each gene and the bars below the gene indicate the frequency of isolation of mutant strains expressing the gRNAs. (**c**) Developmental defects in recreated CRISPR mutants. Aggregation-less mutants are indicated using arrowheads. Scale bars, 1 cm.

Individual *Dictyostelium* cells aggregate in response to starvation to form multicellular fruiting bodies. To isolate mutants that exhibit developmental defects and identify the genes responsible for the phenotype, CRISPR mutant pools were generated from genome-wide and kinase sgRNA plasmid libraries (Fig. 4a). Culturing the mutants on bacterial plates yielded 21,489 clones for phenotypic observations. Since the number of mutants was less than the total number of designed gRNAs, saturation of mutagenesis was not expected. Nevertheless, 280 clones that exhibited various phenotypes were isolated, such as aggregation defects, developmental delays and abnormal fruiting bodies. Analysis of the target sequences of the CRISPR vector in the mutants revealed gRNA sequences in 260 (92.9%) of the 280 clones, with each clone containing an average of 1.51 different gRNAs (Supplementary Data 4). The presence of multiple gRNAs in some of the clones was probably because of the pooled electroporation of CRISPR stable expression vectors, which resulted in the simultaneous expression of multiple CRISPR vectors in some clones. Of the 309 genes, 33 (10.7%) appeared multiple times in independent clones, including some that had independently mutated in multiple regions of the same gene with common phenotypes (Fig. 4b and Supplementary Table 4). The top two genes, *yakA* and *pkaC*, in which gRNAs appeared independently > 10 times, are essential for cell aggregation^43,44^. The third gene, *tor*, does not have any gene knockout mutant isolated via homologous recombination; however, it has been suggested that mutants with an amino acid substitution generated by the chemical mutagen NTG present with aggregation defects^24,45^. Moreover, indel mutations at the target site were observed in 14 (93.3%) of the 15 selected clones, indicating that the designed CRISPR system was highly efficient (Supplementary Fig. 4). To confirm that the gene mutations induced the observed developmental defects, 16 genes were selected to recreate CRISPR-mediated mutants. A mixture of the transformants was plated on bacterial plates to observe the developmental phenotype. Based on the results, three genes (*yakA*, *pkaC* and *tor*) were associated with strong aggregation defects and eight other genes were related to aberrant development (Fig. 4c and Supplementary Table 5). In summary, these results demonstrated that these procedures allow the identification of genes responsible for the target phenotype.

### Broad-spectrum mutations and various phenotypes of the *tor* mutant

**Figure 5:**
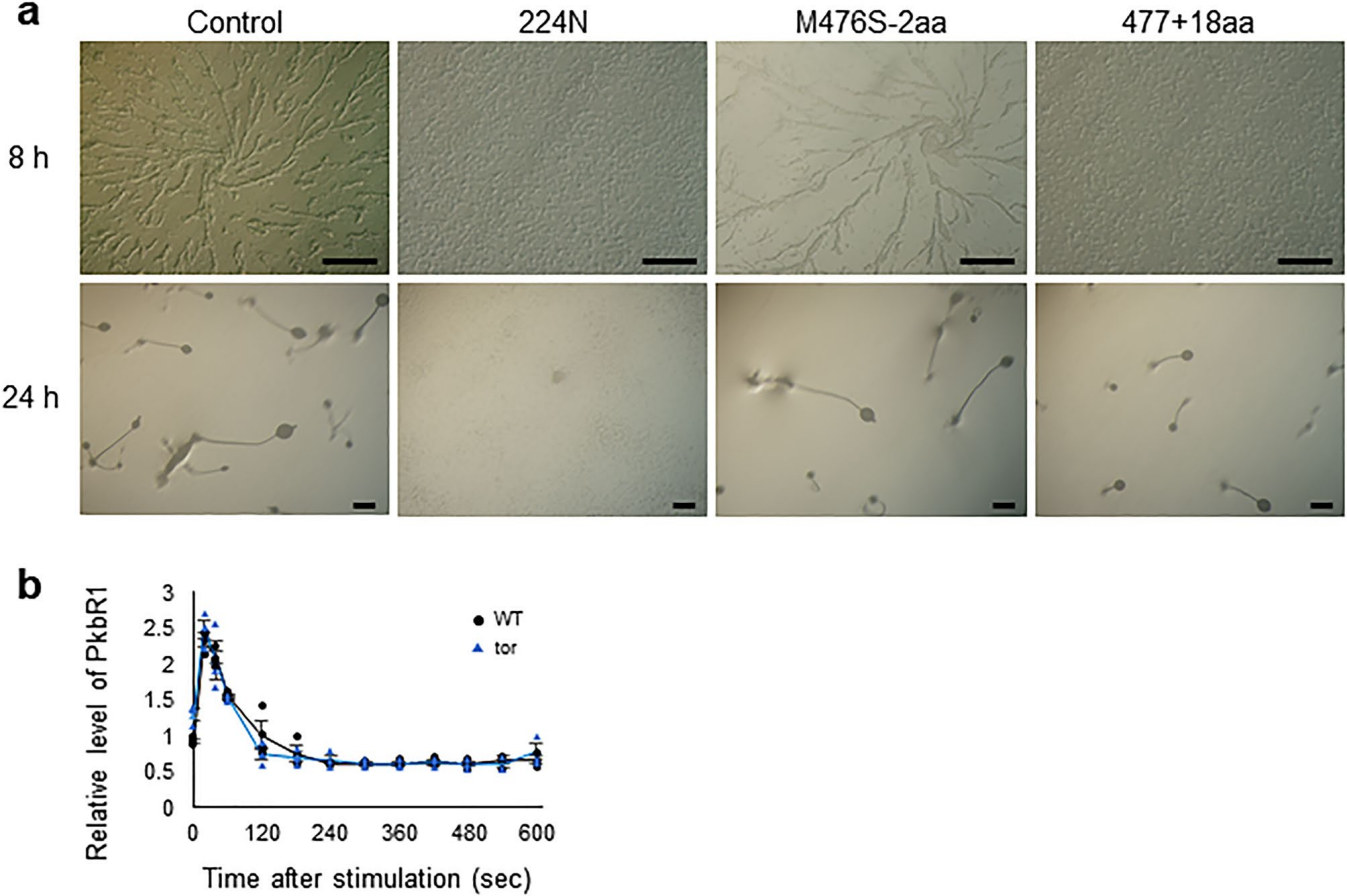
In-frame indel mutations in *tor* identified in the screens. (**a**) Developmental phenotypes of in-frame indel mutations in *tor* cells. The cells were plated on non-nutrient agar and imaged. Scale bars, 250 µm. (**b**) Chemoattractant-mediated activation of PKBR1 in 224 N *tor* mutants. Phosphorylated PkbR1 was analysed by immunoblotting and the band intensity was quantified. Mean values from three independent experiments and individual data points are shown. Error bars represent the SEM.

Phenotypic screenings were conducted, and genome-edited mutants of *tor*, for which gene knockout by homologous recombination had not previously been achieved, were isolated from both screens. The *tor* mutants were all caused by in-frame indels and no gene knockout by frameshifts were observed (Supplementary Fig. 3 and 4), suggesting that this gene is essential, which is in accordance with previous reports^45^. Studies of *Dictyostelium* have indicated that TOR (target of rapamycin) complex 2 (TORC2) is involved in chemotaxis and cell migration via phosphorylation of protein kinase B (PKB)^46–48^. The Tor protein structure contains 11 HEAT repeats and flexible inter-unit loops in the range of 266–1,249 amino acid residues, where the components of the TORC2 complex Pia and Rip3 are presumed to interact and are required for TORC2 integrity. Aggregation defect mutants exhibited point mutations in the loop sequence before the first or between the first and second repeat sequences. A recreated CRISPR mutation with an asparagine residue inserted at position 224 (224 N) exhibited clear aggregation defects, whereas aggregation streams were observed in a mutant with a single amino acid substitution at residue 476 and deletion of two amino acids (M476S-2aa) (Fig. 5a). However, downstream phosphorylation of PKB occurred in this mutant in response to stimulation by cyclic adenosine monophosphate (cAMP), suggesting that the interaction with the TORC2 complex was not completely abolished after cAMP stimulation (Fig. 5b). Similarly, an insertion mutant of 18 amino acid residues at position 477 (477 + 18aa) displayed growth defects (Fig. 3c), while the M476S-2aa mutant exhibited normal growth. The isolation of a broad-spectrum of mutations, which could not be easily isolated using the conventional knockout method using homologous recombination or tag-insertion by the REMI method, demonstrates the power of this technology as a genetic screening method to better understand gene function.

## Discussion

A CRISPR-based genetic screening system for *Dictyostelium* was developed to identify the genes responsible for a particular phenotype. The proposed method provides a wider range of mutations, with highly efficient genome-editing vectors for the screening and analysis of novel genes that have not been isolated to date. This method is especially effective for the acquisition of various mutants, including frameshift and in-frame indels, which are relatively difficult to isolate using conventional methods, such as REMI, antisense RNA and RNAi.

Important considerations for the success of genetic screening include the editing efficiency of the CRISPR system and saturation of mutagenesis. Deep-sequencing analysis of four on-target genes revealed that the newly developed all-in-one CRISPR/Cas9 stable expression vector was more efficient than conventional transient expression vectors. Analysis of gRNA of the individual developmental mutants also revealed that 15 (93.8%) of the 16 identified gRNAs had functionally targeted the loci. Although the efficiency varied with the design of the gRNAs, the editing efficiency was sufficiently high compared with other screening systems in various organisms^49–52^. Therefore, the proposed conditions are suitable for the genome-wide screening of *Dictyostelium*. These methods are also suitable for saturation of mutagenesis. As a conventional screening technique, REMI can be used to obtain thousands of clones via one round of electroporation^12,53,54^. In contrast, one round of CRISPR electroporation can provide approximately 5 × 10^5^ mutants containing approximately 97% of the designed 27,405 gRNAs for genome-wide screening. Thus, in a typical procedure of transformation, multiple electroporations can saturate a targeted gene with mutations and allow the genetic screening of several independent mutant strains exhibiting a common phenotype.

Since numerous mutants can be easily generated by CRISPR libraries, strategies to cover large-scale mutant pools during selection are important for successful screening. As evidenced by the growth selection process, methods that allow the selection of millions of cells on a plate are especially effective. However, when selecting developmental defects, only approximately 20,000 clones are analysed; therefore, the majority of CRISPR mutants generated by a single electroporation are excluding. Moreover, tenfold more plates must be prepared to analyse tenfold more mutants; hence, it is important to improve the efficiency of selection methods. For selection of REMI and NTG-treated mutants in *Dictyostelium*, various powerful strategies were applied, including adhesion selection on 10-cm plates, electrotaxis experiments with barcoded microplates, high-throughput growth and development assays that affect lithium and valproic acid, and counterselection with 5-fluoroorotic acid to enrich genes that suppress polyglutamine aggregation^28,54–56^. Because these techniques allow for the screening of numerous cells, they are easily adaptable to CRISPR mutant pools with millions of mutants. In addition, it may be useful to create CRISPR mutant pools in cells expressing a reporter system, such as fluorescent proteins, and to use flow cytometry to select cells exhibiting specific responses. This method allows us to select from millions of nearly saturated mutations and the identification of candidate genes using this method is expected to cover almost all variants with the desired phenotype. Indeed, the effectiveness of this technique has been demonstrated in several mammalian cell lines^57–59^. Further, it should also be noted that knock-in of fluorescent proteins can also be performed with high efficiency using the CRISPR system in *Dictyostelium* to equalise the number of the reporter copies among individual cells^23^.

Several studies have reported that off-target cleavage by CRISPR/Cas9 depends on the gRNAs to some extent^39,60,61^. The present study suggests that this rule for characteristic gRNAs also applies to *Dictyostelium*; therefore, well-designed gRNAs would allow for genetic screening with almost no concern about off-targets. In particular, it is unlikely that two different gRNAs targeting the same gene show a common phenotype and have similar off-target cleavage sites. Thus, for selecting them from among several million clones, such as using the growth assay, an off-target sample can be ignored because data on the under- or over-represented gRNA are derived from several hundred independent clones on average. For the isolation of individual clones, such as through developmental assays, a cut-off of at least two different mutant hits per gene was used to select candidate genes for further validation. However, a cut-off of a minimum of three hits can reduce false positives including the effect of off-targets. All six selected genes, with a minimum of three hits, reproduced developmental abnormalities, supporting the idea of the cut-off. In contrast, there remains the possibility that true genes, with only hit once, could be truncated. In the developmental mutants, 89% of genes had only one hit, and validating all by regenerating CRISPR mutants is especially time-consuming. An alternative approach is the amplification of sgRNA regions from these mutants to recreate secondary sgRNA libraries and perform further selection to narrow the field of candidates. However, it is also important to note that CRISPR vectors extracted from mutants may contain nucleotide substitutions to scaffold RNA and/or tRNA sequences that regulate sgRNA expression.

Genome-wide and kinase CRISPR libraries, including sgRNA plasmids and mutant pools, were deposited in the stock centre of the National BioResource Project Nenkin (https://nenkin.nbrp.jp/), and are available to all researchers. Storage without confirmed genetic modification was not considered because individual mutants can be easily recreated using the CRISPR vector as demonstrated in this study. Although the genome-wide collections of REMI mutants and pGWDI plasmids for mutagenesis are obviously useful resources in terms of their coverage and extensive use in research on *Dictyostelium*^25^, CRISPR libraries have several advantages over existing mutant resources. First, the range of mutations derived from one gRNA is wide and includes frameshift and in-frame indels, thus increasing the possibility of obtaining the desired phenotypes, as revealed in this study with *tor* mutants. Second, as the identification of candidate genes only requires sequencing analysis of the gRNA in the CRISPR vector, standard molecular biology techniques are sufficient and no specialised machinery or technology is required. Third, it is easy to recreate mutants once the candidate gRNAs are identified; therefore, this technique is effective for phenotype validation. This is also true for the recreation of mutation pools, where the CRISPR sgRNA plasmid library can be obtained from the stock centre and mutation pools can be created using any target cell line. Here, we present a genome-wide CRISPR library of *Dictyostelium* and describe methods that can provide a promising platform for genetic screening.

## Methods

### Construction of the CRISPR/Cas9 stable expression vector

An all-in-one CRISPR/Cas9 vector that stably expresses Cas9, sgRNA and the blasticidin-resistance gene was created. In brief, a tRNA-sgRNA cassette containing two Esp3I sites was inserted into the extrachromosomal vector pDM326^62^ via the XhoI and HindIII restriction sites to generate the vector pTM1423. NgoMIV restriction sites flanking the Cas9-NLS expression cassette was subsequently cloned into the NgoMIV site of the vector pTM1423 to generate the vector pTM1369 that stably expresses Cas9 and sgRNA (Supplementary Fig. 1). As the position and direction of the Cas9-NLS and tRNA-sgRNA cassettes appear to affect genome-editing efficiency^30^, the Cas9-NLS, tRNA-sgRNA and blasticidin-resistance cassettes were obtained from a pTM1285-derived vector and cloned into HindIII- and BamHI-digested vector pDM304 to generate the vector pTM1709 (Supplementary Fig. 1). The plasmids were deposited in the stock centre of the National BioResource Project Nenkin.

### Designing custom sgRNA libraries

Target sequences for the genome-wide sgRNA plasmid library were computationally designed as previously described^63^, but with minor modifications. The protein-coding nucleotide sequence of *Dictyostelium* was obtained from the Ensembl Protists genome-centric portal (https://protists.ensembl.org/), and a list of potential gRNAs was generated within the window of + 1 to + 400 bp relative to the start codon using Python scripts. An additional N sequence was inserted into the exon-exon junction to avoid selecting an inappropriate target sequence that spans the exon–intron boundary. To design gRNAs with fewer potential off-target sites, the scripts were executed with the following optional parameters: maximum number of each gene, 3; minimum spacing between cleavage sites of gRNAs, 10 and minimum GC content, 20. Finally, a maximum of three gRNAs were obtained, and pseudogenes and the duplication region of chromosome 2 were excluded to generate a genome-wide library (Supplementary Data 1). To design the kinase sgRNA plasmid library, standalone editions of Cas-Designer (http://www.rgenome.net/cas-designer/), Cas-OFFinder bulge (https://github.com/hyugel/cas-offinder-bulge) and Cas-OFFinder Binary ver. 2.4 (https://github.com/snugel/cas-offinder) were downloaded and installed on a Mac OS X graphical operating system with Python 3.88 (https://www.python.org/downloads/release/python-388/). Genome sequences of *D. discoideum* were downloaded via Ensemble Protists and converted to a 2-bit format using the Anaconda package ucsc-fatotwobit (https://anaconda.org/bioconda/ucsc-fatotwobit) to expedite the process. Of the 12,321 protein-coding genes, 12,126 (98.4%) were selected as targets. Genes that were not currently registered with dictyBase (http://dictybase.org/) and extrachromosomal genes were excluded. Sequences of gRNAs within the coding region were designed using Cas-designer with reference to the data on the exon–intron boundary of each gene on dictyBase. Output data were filtered as follows: exclusion of TTTT stretches (Pol III termination signals), GC content > 20%, out-of-frame scores > 66, and a mismatch number 1,0,0. For the duplicated genes within chromosome 2, mismatch numbers 2,0,0 were used (Supplementary Data 2). The target sequences were tiled within the kinase genes by narrowing down the list of whole genes to 283 kinase genes and further selecting five target sequences per 1 kb. If the filtering conditions were too stringent to ensure sufficient targets, the sequences with the next highest GC content and out-of-frame scores were considered as additional candidates. In contrast, the mismatch number increases the risk of off-target, so the conditions were not relaxed. In addition, 100 non-target controls with no similar sequences in the *Dictyostelium* genome were added (Supplementary Data 3).

### Designing gRNAs with mismatches and validation of CRISPR/Cas9-mediated mutations

The Python script Cas-Designer was used to design potential off-target sequences with mismatch numbers 1,1,0. This score indicates that the on-target region is 100% matched with the genome sequence and a single-nucleotide mismatch of 20 nucleotides in an off-target region present in one locus in the genome. gRNAs with a single-nucleotide mismatch inside or outside of the seed region, which is important for the specificity of the target, were selected. The 20-nucleotide sequences were then synthesised as an oligonucleotide pair with the overhang sequences AGCA and AAAC, respectively (Supplementary Table 6). Annealed oligos were cloned with a Golden Gate assembly into the all-in-one CRISPR/Cas9 vectors pTM1599, pTM1668 and pTM1709 as described previously^23^ (Supplementary Fig. 5). A list of CRISPR vectors for the off-target assay is presented in Supplementary Table 7. To confirm the induction of on- and off-target mutations by transient or stable expression of SpCas9 or SpRY, gDNA was extracted from cell populations in culture dishes to produce amplicons for next-generation sequencing (NGS) (Supplementary Fig. 6).

### Generation of a custom sgRNA library

Designed target sequences for kinase genes (Supplementary Data 3) were synthesised using array-based technology (CustomArray Inc.) in duplicate to increase the oligo uniformity and decrease the dropout rates. In contrast, due to the large number, the genome-wide targets (Supplementary Data 1) were synthesised only once. The overhang sequences were appended to the ends of each oligonucleotide for PCR amplification, which was performed using ~ 28 ng of pooled oligonucleotides as a template, the primer pair sgRNApool_Fw (5′-AATAACGCCACGGTCGCAGGTTCGATCCCTGCATCGAGCA-3′) and sgRNApool_Rv (5′-CTTATTTAAACTTGCTATGCTGTTTCCAGCATAGCTCTTAAAC-3′), and KOD -Plus- Neo DNA polymerase (TOYOBO) in a final volume of 50 µL. PCR conditions were as follows: 10 s at 98 °C, 10 s at 63 °C and 15 s at 68 °C for 20 cycles. The resulting 103-bp PCR products were purified using a FastGene Gel/PCR extraction kit (Nippon Genetics) and cloned into the Esp3I (Thermo Fisher Scientific) pre-digested vector pTM1369 (for the genome-wide sgRNA library) or pTM1709 (for the kinase sgRNA library) using a NEBuilder HiFi DNA Assembly kit (New England Biolabs) (Supplementary Fig. 5). The assembled product was precipitated with isopropanol and electroporated into Endura Electrocompetent Cells for CRISPR (Lucigen). The transformants were grown at 37 °C for 12–14 h on LB plates with 100 µg/mL of ampicillin. Individual colonies were gently scraped off using a cell spreader and plasmid DNA of the CRISPR library was prepared using a NucleoBond Xtra Maxi kit (MACHEREY–NAGEL). The resulting pooled plasmid DNA (pTM1376; genome-wide sgRNA library, pTM1810; kinase sgRNA library) was aliquoted and stored at −20 °C.

### Cell culture, transformation and gDNA extraction

Axenic strain AX2 cells were cultured at 22 °C in HL5 medium or on SM agar plates with *Klebsiella pneumoniae* (KpGe) as a food source. Transformation was performed as described previously^23^ with minor modifications. Before electroporation, 0.5 µg of the CRISPR sgRNA library was mixed with cells and transferred to an electroporation cuvette. To repair the damage caused by electroporation, the cells were incubated on a 10-cm culture dish containing 10 mL of HL5 medium. To obtain cells expressing Cas9 and sgRNA, the HL5 medium containing 10 µg/mL of blasticidin S was replaced after 8–24 h and the cells were further cultured for another 4–6 days. The resulting mutant libraries were used for further phenotypic screening or gDNA extraction for use in an off-target assay and genetic screening. gDNA was extracted from approximately 3 × 10^6^ cells. For the transient expression of Cas9 and sgRNAs for the off-target assay, cells were transformed with 10 µg of the all-in-one CRISPR/Cas9 vector. After electroporation, the cells were rescued by culturing in the HL5 medium for 8–24 h and maintained for another 1–2 days in fresh HL5 medium containing 10 µg/mL of G418 to induce expression of Cas9 and sgRNA. Cells were cultured for another 5 days without G418 and gDNA was extracted from the growing cells for Illumina sequencing by two methods: a simple method using ProK treatment and a Wizard Genomic DNA Purification Kit (Promega) in accordance with a previously described method^23^ and the manufacturer’s instructions, respectively.

### Selection for growth and development

The kinase sgRNA library was electroporated into AX2 cells to obtain three independent mutant pools of approximately 5 × 10^6^ cells each. These cells were seeded on 10-cm tissue culture plates and subcultured every 2–3 days to maintain ~ 80% confluency. Each plate was grown for approximately 37 generations (14 days). gDNA was isolated using a Wizard Genomic DNA Purification Kit before and after selection and processed for Illumina sequencing (Fig. 3A). For isolation of developmental defective mutants, mutant pools expressing the sgRNA plasmid library for the kinase or the genome-wide library were plated onto SM agar plates with bacteria as a food source at a density of ~ 1,500 cells per 245-mm square dish and allowed to develop for 4–7 days. Plaques exhibiting developmental defects were isolated and transferred to 96-well plates containing HL5 medium with 10 µg/mL of blasticidin S. To repeat the phenotypic assay for another round of selection, growing cells in the wells of 96-well plates were gently washed once with KK2 buffer (16.2 mM KH_2_PO_4_ and 4.0 mM K_2_HPO_4_) and the cell suspensions were then transferred into fresh 96-well plates containing SM agar with bacteria as a food source (Fig. 4A). The mutant strains, which again showed developmental abnormalities, were transferred into 96-well plates containing HL5 medium and gDNA was isolated from fully grown cells by the ProK treatment method.

### Preparation of DNA for amplicon sequencing

To analyse sgRNA in CRISPR/Cas9 vectors, PCR amplicons were prepared through two separate PCR assays using primers containing index barcodes (Supplementary Fig. 7). The barcoded amplicons were submitted for secondary PCR and an Illumina index was added. For the analysis of mutations introduced to the on-target and off-target loci, gDNA was extracted and amplified using primers containing index barcodes (Supplementary Table 8 & Supplementary Fig. 7A). The barcoded amplicons of each replicate were pooled and submitted for secondary PCR. The PCR products were sequenced with the MiSeq System (Illumina), which yielded 250-bp paired-end reads aiming for > 2,000 reads per individual mutant pool. To determine the distribution of gRNA in the kinase and genome-wide gRNA libraries, pooled plasmid DNA (pTM1376 and pTM1810) was amplified by PCR with appropriate primers (Supplementary Table 9 & Supplementary Fig. 7B). The PCR products (~ 170 bp) of each library were pooled and sequenced with the NovaSeq System (Illumina), which yielded 150-bp paired-end reads aiming for > 100 reads per individual gRNA. For analysing the CRISPR mutant pool before and after selection, gRNA insertions were amplified from the gDNA using appropriate primers (Supplementary Table 10). Equal amounts of the PCR products (~ 110 bp) were pooled, purified using a Gel/PCR purification kit, and sequenced using the NovaSeq Sequencing Platform (Illumina), which yielded 150-bp paired-end reads aiming for > 500 reads per gRNA. For analysis of individual CRISPR clones obtained through the developmental phenotype assays, gDNA was amplified using primers specific to each position in a 96-well plate (Supplementary Table 11). These primer sets comprised 16 forward primers and 24 reverse primers to allow up to 384 samples to be pooled and sequenced with the MiSeq System (Supplementary Fig. 8). An equal amount of the PCR products (~ 230 bp) from each clone was pooled and sequenced with the MiSeq System, which yielded 250-bp reads aiming for > 100 reads per clone.

### NGS data analysis

Raw NGS data were analysed using Python scripts to calculate the read counts in each barcoded group. In brief, FASTQ files were extracted, the index barcodes were identified, and target sequences were counted. For the off-target assay, FASTQ data were analysed through CRISPResso2 (https://crispresso.pinellolab.partners.org/)^64^ and the modified and unmodified genome loci were calculated. For analysing of the sgRNA plasmid library and mutant pools, differential sgRNA distributions were analysed using the MAGeCK pipeline^42^. To identify significant genes for the growth selection, Day 14 mutant pools were compared with those of Day 0. The RRA method was used to compare the fold-changes of sgRNAs of each gene before and after selection. GO term enrichment analysis of genes associated with growth selection was performed using a web tool^65,66^. For the analysis of sgRNA obtained from developmental mutants, FASTQ data were converted to FASTA files and index barcodes were identified to mark the positions on a 96-well plate grid (Supplementary Fig. 8).

## Supplementary Information

Supplementary Information.

Dataset S1.

Dataset S2.

Dataset S3.

Dataset S4.

Supplementary Table 2.

## Data Availability

All data are presented in the manuscript or the supplementary materials. The plasmids and cell lines generated in this study will be available from the National Bioresource Project ‘Cellular Slime Molds’ (NBRP Nenkin).
