# Supplementary material for: CRISPR/Cas9-based genome-wide screening of Dictyostelium
Source: Sci Rep. 2022 Jul 2;12:11215. doi: 10.1038/s41598-022-15500-3 (PMC9250498; doi:10.1038/s41598-022-15500-3)
Supplement: Supplementary file 1 — Supplementary Information. [file 41598_2022_15500_MOESM1_ESM.pdf]

CRISPR/Cas9-based genome-wide screening of *Dictyostelium*

Takanori Ogasawara[1], Jun Watanabe[1], Remi Adachi[1], Yusuke Ono[1], Yoichiro Kamimura[2], Tetsuya Muramoto[1]*

[1]Department of Biology, Faculty of Science, Toho University, 2-2-1 Miyama, Funabashi, Chiba, 274-8510 Japan.

[2]Laboratory for Cell Signaling Dynamics, RIKEN, Center for Biosystems Dynamics Research (BDR), Suita, Osaka, 565-0874 Japan

*Corresponding author:

tetsuya.muramoto@sci.toho-u.ac.jp

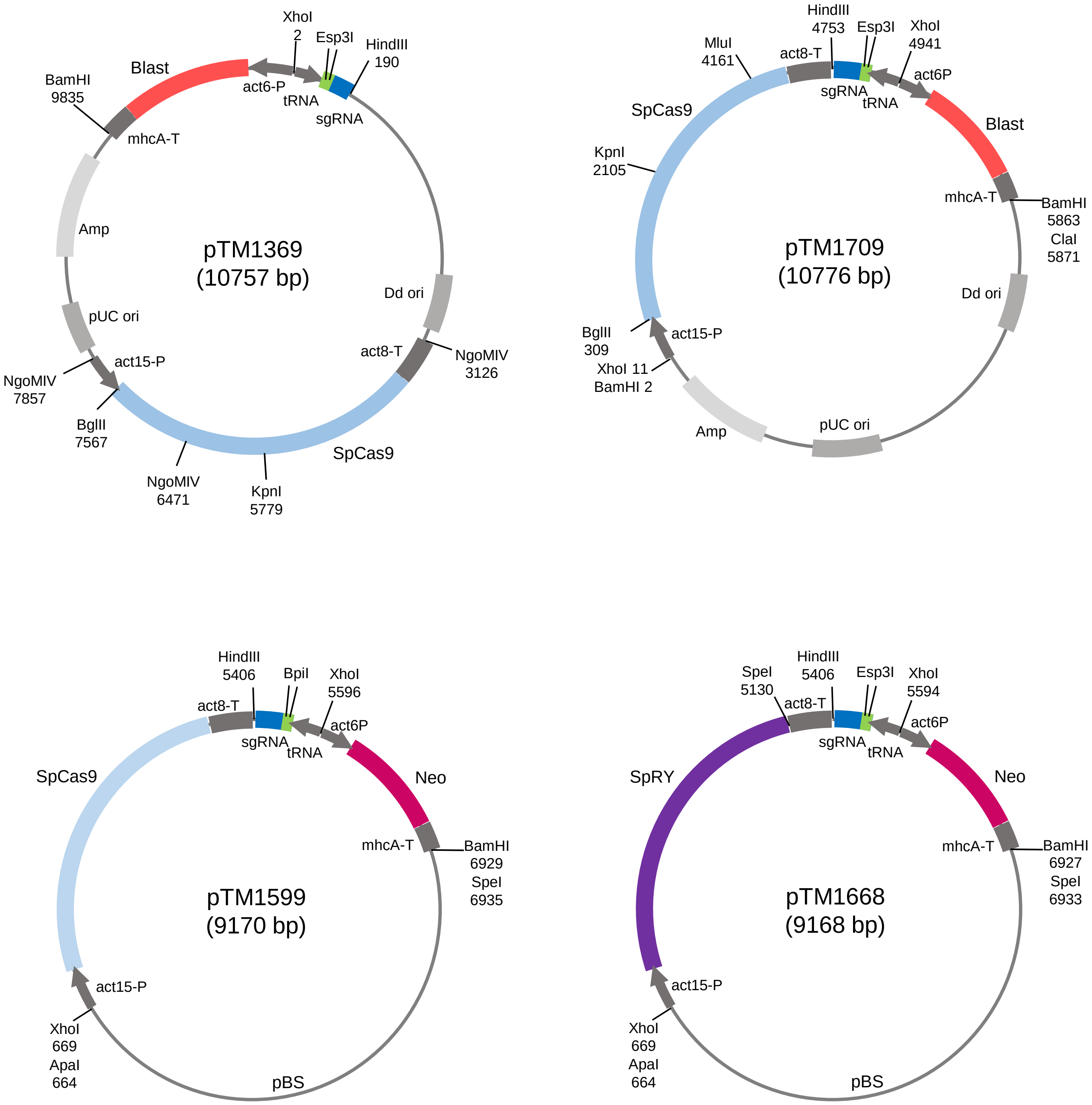

Supplementary Figure 1 Restriction maps of all-in-one CRISPR/Cas9 vectors and predicted DNA sequences.

pTM1369, a stable expression vector containing blasticidin-resistance cassette. SpCas9 expression module was inserted in the NgoMIV site. pTM1709, a stable expression vector containing blasticidin-resistance cassette. SpCas9 and tRNA-sgRNA expression cassettes were arranged next to each other. pTM1599, a transient expression vector containing neomycin-resistance cassette. pTM1668, a transient expression vector containing neomycin-resistance cassette. SpCas9 variants SpRY was used to recognise relaxed PAM sequences.

```
>pTM1369
CTCGAGGCTCGATTAGCTCAGTCGGCAGAGCGTGGTGCTAATAACGCCACGGTCGCAGGTTCGATCCCTGCATCGAGCAGGAGACGGGCGTCTCGGTTTAAGAGCTATGCTGGAAACAGCATAGCAA
GTTTAAATAAGGCTAGTCCGTTATCAACTTGAAAAAGTGGCACCGAGTCGGTGCTTTTTTTAAGCTTATCGATTTCGAAAAAGTAAATAGCAATTATTACAAAATTCAATCCGAATCTACCCAAATA
AATTCCAATGAAATTGCCGATTTAAAAAAGTTTATTAAAGAAGAAGTCAATAAAACTTCTTCCAAAATTGATTTCTTTTTAGTTTCTTCAACAGATGCCCTTTCAAATCCAGAAAATTATTCTCTCT
TAGAAGTAAAGTGTATTAATTGTCATTCTTTGTGTCAAGGAAAAAATTTATATATTTCATGTACAAGAGATGGATGTCAAAACAATATTTGCTATAATTGTTTAGGAATAAACATAAACATATATAA
TGTTGTTATTAATTCTAAACTTTGCCCTCCATGTTTCAATGATTCGGTAATCAACAAGAAGTGTGCCATGTGTAGTAAGAACGGAACTAAATGTAATTTGAACCAAGAATGTAAACTTCATCTTTGT
GCACAGTGTTCTAAAAAGTGTCTATACATTCTGAGAGTCAAAACTAATTAAATAAAATATAAACTTAATTTCTAAATAAACTCATTTAAAAATATTTAAATAATATGAATTTATAACTGTAATTATT
GTATTAAAAAATTATATAATTATTTAATGTTAAAAATGTATTAAAATAATTATAAAAAAATATAACAAAAATTTTCGTAAAAATAATTTGTAAAAAAGCTATTAAAAATATTATGAAAAAAAAATTA
AAAAAATTATTAAATTGTTTTTGTAATTAAGCTATTAAAATAATTATAAAAAAAAAATTTTTAAAATTTTAAAAATATTTTTTGTAAAAAAGTATTAAAATAATTATGAAAAAAAAATTTTCTAAAA
AATTAAAAAAAAAAATTAAAATATATTTTATGTTAAAAACGTATTAAAATAACTATTAAAAAAATTATATTTAAAAAAGTATTAACTTTTTTTTAGGTGTGGTTGTGGGGTGGGGTTTAATATATTAT
AATAAAAAATTATTTTTTGTTCATTTATTATTTTCATTGTATATAATGTACTCAACAACGTTATTATTTTTTCTTTTTTTTTTTTATTGTATCAAAATCTTCTGTTCTTCAAAATGATCAGATTGAAG
TAAAATATTTTCAACTTCTTATTGTTATGTATCAAAAAGAAAACTGTGTTGAAAAGTCAATGACAGGCGCCGTAATTTATGATGAATGTAATATTCATGGAAGAGTTGAAACAAATAGTACTCATGC
GCTTTTTTATGATGACATTGAAACAAATAATTCAAGATGTAACAATTTTCGTAATTTAACAAACTTAATTAAACTTAATGAATGTATTAATGACGAGTTTGGAGAGTCTATTCTTTATAAAGAATAT
AATGAAACTGATGATGGTTATTTGTTTAGAGTGGAAGACAGCTTTGTTGAAATTACTTCTCTTTCAATGGATTGTACAAAAAATAGTAAAACAATTATTGAAAAATTCAACATTTGTTCAAAATTTG
AAAATGTATATCATATTACAAACATTACACAAGAGAAATCCAATAGATTTACATGTACAGATCCATTGTGCCACTATTGTAAGAATGAAAACATTCAAAACAATCTTGATTTTAAAACAACAAAGTG
TACTCCAAAGTATGGTGCATCTGATTCTGAATTTTTATCAACAATTTACAATCCAAAGCTCGATGGCTCAAATAACGGTATGGAAAAGTCAGTAACTCAAGAAAAAAACATTTCAAATAATTTAAAA
ATTAATATATATTTAATTTTCTTTTTAATTATTTTTTTAATTAAATAAAGTTTTATTATTTTTTAAGAGTAATTATTGCTCTTTTTTCATTTGAAACACCAGAAGCTAAACGTAATTGTTGTTGACT
GAAATTTTTTATTTTTTTTGGGGTAATAGGATTTCCTTTTTTATGAAGATTAATATCTTTGACTCGTGAAACATTCTTTTTAACTTTTGTTTTTTCTGTTGGTTTATCATTTGTTTTTTCACTAATT
TCAATACCATCTTGACGTTCATTCATAACTTCATCTTTTTTTTTTCCTGTTTCTGTATCTTCTTCTATTTTTTTTTTCTTTATCTTTTTCTTTATCTTCTTCTTGTTCTTCCTCTTCTTTTTCTTCTT
CTGATACTGCAGGTGTTTCTTCTTCTTCTTCTTCCGATATTGTCGGTTTTTCTACTTCTTCTTCTTGTTCTTCTTCTTCTTCTTCTTCTTCTTCTTCTTCTTCTTCTTCTTCTTCTTCTTCTTCCGGTAA
TTTATTAATTATATTTCTTTTTTTATATGAATTACGTTTGGTTTGTGCAGTAATTTCCTTACATAGAGTGCAGCTTTCAAGAAAAAATTTCAATTTCTTCGTTTGTTGCATAATAACCACTGTCTTTG
ATATGATTAAACATTTTTGATTTTCTTAAATGCTTTCCTTCTTTAATATGAAAATTATCGAATTCTAATTCATTAAGAACAATAAGCTCCCCTAATTTAAAAAATTAGTTAAAATAAATTAAAATGA
ACATGTATAAAGATGGATTTTACCATTTTTTGAAATTCTAAATAACTTTTCTTCATCTCCAATCTTTTTGACTGAAAAACGATTTTTAATTGAAGTTATTGTTCTGTGAGTGTTTTGAATCGCCCAT
TTCTCTAAATCAGTTTGAGATAGTGTTTTATAATCTGAATTGTTATACACAACTTTTGCTCTATTAACCAAATATTTAAAGATTTCATCATCAACTGAATATTTTGACTTTACGATTCTTGTCCAAA
AAACAATTTCTACTACTATCATTTTTTATTTATAAAATAATTTAAATACAAAAATGAATTTTTTTTTTTTTTAAAAAAAAAAAAAATTTGAAAAAAAAAAAAAAAAAAATTTTAAAAAAAAAAAAAAAAA
AAAAAAAAAAAAAAAAATCAAATAAAAAGTAAAAAATAAAAACCGAAAAACATTCATTGTAATTTCAAATGTCGAGGCCGGCTATCTTTTTGATTTTCATACTCTTTGGCTGTTTTACATCTAATGC
ATATATTTATCTTCACCATTCAAAATGTATCTGTAAATAATAAATTTTTTAAATATATTTGATTTTTTTGATAGAAAAAAAAAAAAAAAAAAAAAAAAAAAAAAAAAAAAATTAAAATAACTTAATAAT
TATTAAGATTTAAAAAAAAAAAATTAAAAAAAAAAGAAAATATTAGATTATTACAACAATTTGTTTTTTTATTATTTATTAAATAATTTATTTATTTAACTAGCGATGGAACTACCTACCTTGCGCT
TTTTCTTGGGAGCTCCCTCATCCCCTCCGAGCTGTGAGAGGTCAATCCGAGTTTCGTAAAGTCCGGTGATGCTCTGGTGGATGAGGGTGGCGTCCAGCACTTCTTTGGTGGAGGTATAGCGTTTCCG
GTCGATGGTGGTGTCGAAGTACTTGAATGCGGCAGGGGCACCAAGATTGGTGAGGGTGAACAGGTGGATGATATTCTCAGCCTGCTCCCGAATTGGTTTGTCTCTGTGCTTATTGTATGCGCTAAGG
ACTTTATCGAGGTTGGCATCAGCGAGAATGACGCGTTTAGAAAACTCGGAAATCTGCTCAATGATCTCGTCCAGATAATGCTTATGCTGCTCCACAAACAGTTGCTTTTGCTCGTTGTCCTCGGGGC
TCCCCTTCAGCTTCTCGTAATGGCTTGCAAGGTAGAGGAAATTCACGTACTTGGAGGGGGAGAGCCAGCTCATTACCCTTCTGAAGTTCGCCAGCGGAGGCGAGCATCCGCTTCCGCCCATTCTCCAG
TTCGAACAGGCTATACTTTGGGAGCTTGATGATAAGGTCCTTCTTGACTTCCTTGTAACCTTTGGCTTCGAGAAAGTCAATTGGGTTCTTCTCGAAGGAGCTGCGCTCCATAATGGTGATACCCAGC
AGTTCCTTCACGCTCTTGAGCTTCTTAGACTTTCCTTTTTCCACCTTAGCCACGACCAGGACAGAATAAGCCACAGTTGGTGAATCGAATCCGCCGTACTTCTTAGGGTCCCAATCCTTCTTCCTTG
CAATGAGCTTGTCGGAGTTGCGCTTGGGGAGGATTGATTCCTTAGAAAAGCCTCCGGTTTGCACCTCGGTTTTCTTCACGATGTTCACTTGAGGCATAGAAAGCACTTTGCGCACGGTAGCGAAATC
TCTGCCCTTGTCCCACACGATTTCGCCAGTCTCCCCATTAGTCTCGATGAGCGGCCTCTTCCGGATCTCACCGTTTGCAAGGGTGATTTCGGTCTTGAAGAAATTCATGATGTTGCTGTAGAAGAAG
TACTTTGCGGTGGCCTTTCCGATCTCCTGCTCAGATTTGGCAATCATCTTGCGCACGTCGTAGACCTTGTAGTCTCCGTACACGAACTCGCTTTCCAGCTTTGGGTACTTCTTGATCAGGGCGGTTC
CCACCACGGCATTCAGATATGCGTCATGAGCATGATGATAGTTATTGATCTCCCGCACTTTGTAGAATTGAAAGTCCTTTCTGAAATCGCTGACCAGCTTGGACTTCAGGGTAATCACCTTCACCTC
GCGGATAAGTTTGTCGTTTTCGTCGTACTTGGTATTCATGCGTGAGTCAAGGATTTGGGCGACATGTTTAGTAATCTGCCGAGTCTCCACAAGTTGCCGTTTAATGAATCCTGCTTTGTCCAGTTCG
CTCAGTCCGCCCCTCTCAGCCTTAGTGAGATTGTCGAACTTCCGCTGAGTGATCAGCTTTGCGTTCAGAAGCTGGCGCCAATAGTTCTTCATCTTTTTCACGACTTCCTCTGAGGGCACGTTATCTG
ACTTTCCCCGGTTCTTGTCTGAGCGAGTCAGCACCTTATTGTCAATTGAGTCGTCTTTGAGGAAGCTCTGAGGGACGATATGGTCCACGTCGTAGTCTGAGAGCCTGTTGATATCAAGCTCTTGGTC
CACATACATATCGCGTCCATTTTGAAGGTAGTACAGATAGAGCTTCTCATTCTGAAGCTGGGTGTTTTCCACCGGGTGCTCTTTAAGGATCTGTGACCCGAGCTCCTTAATCCCTTCCTCAATTCTT
TTCATCCGCTCCCTGCTGTTCTTCTGTCCCTTCTGGGTAGTCTGGTTTTCCCTGGCCATCTCGATGACAATGTTCTCTGGTTTATGGCGACCCATCACCTTGACCAGCTCGTCCACGACTTTCACGG
TCTGGAGAATACCCTTCTTAATAGCGGGTGAACCAGCCAGATTTGCGATATGCTCGTGCAGTGAGTCACCTTGTCCAGACACTTGGGCCTTCTGGATGTCCTCCTTAAAGGTCAGGGAATCATCATG
GATAAGTTGCATGAAGTTGCGATTAGCGAATCCGTCGCTCTTAAGGAAATCAAGAATGGTCTTTCCGCTCTGTTTGTCCCGGATTCCGTTGATGAGTTTGCGGCTAAGGCGTCCCCATCCGGTATAT
CTTCTCCGTTTAAGTTGCTTCATCACTTTATCGTCGAACAGATGGGCGTAGGTCTTAAGCCTCTCCTCGATCATTTCCCGGTCCTCGAACAGAGTGAGAGTCAGGACAATGTCCTCAAGGATGTCCT
CGTTCTCCTCATTGTCCAGAAAATCCTTGTCCTTGATGATCTTAAGCAGATCATGGTAGGTACCCAGGCTTGCGTTGAACCGATCTTCGACCCCGCTAATCTCCACAGAGTCGAAGCATTCAATTTT
CTTGAAATAGTCCTCCTTCAGCTGCTTCACGGTCACCTTTCTATTGGTCTTGAACAGAAGGTCCACAATTGCCTTCTTTTGTTCGCCTGACAGGAATGCGGGCTTCCTCATCCCTTCGGTGACATAT
TTCACCTTGGTCAGCTCGTTGTACACGGTAAAGTACTCGTAGAGCAGGGAATGTTTCGGCAGGACCTTCTCATTGGGGAGGTTCTTATCGAAGTTAGTCATCCGTTCGATGAATGACTGAGCGCTGG
CTCCCTTGTCCACCACCTCTTCGAAGTTCCAGGGAGTGATAGTTTCTTCTGACTTTCTGGTCATCCAAGCAAACCGGCTATTTCCTCTGGCGAGGGGTCCCACGTAGTAGGGGATGCGGAAAGTGAG
AATTTTCTCAATCTTCTCCCTGTTGTCCTTAAGAAAGGGGTAGAAGTCCTCTTGGCGCCGAAGGATGGCGTGAAGCTCCCCAAGATGGATCTGGTGGGGAATGCTCCCATTGTCGAAAGTGCGCTGC
TTCCGCAGCAGATCCTCCCGATTCAGTTTCACGAGGAGCTCTTCGGTGCCGTCCATCTTCTCAAGGATAGGTTTGATAAACTTATAAAATTCCTCTTGGGAGGCACCTCCGTCGATGTAGCCGGCGT
ATCCATTCTTTGACTGGTCAAAGAAGATTTCCTTGTACTTCTCCGGAAGCTGCTGCCGGACGAGTGCCTTAAGCAGGGTCAGGTCCTGGTGGTGCTCATCATACCGCTTAATCATTGAAGCTGAAAG
AGGGGCCTTGGTGATTTCAGTGTTCACGCGGAGAATGTCAGACAGCAGGATGGCGTCAGAAAGATTCTTAGCAGCAAGGAAAAGGTCGGCGTATTGGTCACCGATCTGGGCCAGGAGGTTGTCGAGA
TCGTCATCATAGGTGTCCTTGGACAGTTGCAGCTTGGCATCCTCTGCCAGGTCGAAATTAGACTTGAAATTGGGAGTCAGCCCAAGTGAAAGAGCAATCAGGTTCCCGAACAGTCCATTTTTCTTTT
CACCCGGCAGCTGAGCGATAAGATTCTCAAGTCTGCGGCTCTTTGACAGCCGGGCTGACAGAATGGCCTTGGCATCGACTCCGCTTGCATTGATGGGGTTCTCTTCAAAGAGCTGGTTGTAAGTCTG
CACCAGCTGGATGAACAGTTTATCCACATCGCTATTGTCAGGATTGAGGTCGCCCTCGATCAGGAAGTGGCCTCTGAACTTGATCATGTGGGCCAGGGCCAGGTAGATAAGCCGGAGGTCTGCCTTG
TCGGTTGAGTCCACCAGCTTTTTGCGAAGATGGTAGATGGTTGGGTATTTCTCGTGATAGGCCACCTCGTCGACGATATTACCGAAGATGGGATGCCGTTCATGCTTCTTATCCTCTTCCACGAGGA
ATGATTCTTCCAGCCTATGGAAGAAGCTGTCGTCGACCTTTGCCATTTCGTTGGAGAATATCTCTTGAAGGTAACAAATCCGGTTCTTCCTCCTGGTGTACCGCCGTCTAGCAGTCCGCTTGAGGCG
GGTTGCTTCAGCGGTCTCCCCTGAATCAAAGAGGAGAGCTCCGATCAGATTTTTCTTGATGGAATGCCGGTCTGTATTACCTAAAACTTTAAATTTTTTTGATGGAACTTTATATTCATCTGTAATA
ACTGCCCAACCAACACTATTTGTACCAATATCTAAACCAATTGAATATTTTTTATCCATTTTATTGTTGTATAGATCTGATTTTTATTTTTTATTTAATTTAATTTATTTGTTTTAAGAAATAAGAA
AAAAAAAAAAAAAAAAATCTTTTTATGCAATCTGAAAAAAAAAAAAAAAAAAAAAAAAAAAAAAAAAAAATTTTGAATCCCATTTTTTTTTTTTTAATTTGGGTTTTAAAATTTTCAAATAATAATTAA
CCAACCCAAGTTTTTTAAACCATTTTTTTTTTTAAAGATTTGATGGGATTAATTAATTTGTAATCTATTTAATTCAAAATAAATAAAAATAAATAAAAATTTTTTTTAGCCGGCAGAGGCGGTTTGC
GTATTGGGCGCTCTTCCGCTTCCTCGCTCACTGACTCGCTGCGCTCGGTCGTTCGGCTGCGGCGAGCGGTATCAGCTCACTCAAAGGCGGTAATACGGTTATCCACAGAATCAGGGGATAACGCAGG
AAAGAACATGTGAGCAAAAGGCCAGCAAAAGGCCAGGAACCGTAAAAAGGCCGCGTTGCTGGCGTTTTTCCATAGGCTCCGCCCCCCTGACGAGCATCACAAAAATCGACGCTCAAGTCAGAGGTGG
CGAAACCCGACAGGACTATAAAGATACCAGGCGTTTCCCCCTGGAAGCTCCCTCGTGCGCTCTCCTGTTCCGACCCTGCCGCTTACCGGATACCTGTCCGCCTTTCTCCCTTCGGGAAGCGTGGCGC
TTTCTCATAGCTCACGCTGTAGGTATCTCAGTTCGGTGTAGGTCGTTCGCTCCAAGCTGGGCTGTGTGCACGAACCCCCCGTTCAGCCCGACCGCTGCGCCTTATCCGGTAACTATCGTCTTGAGTC
CAACCCGGTAAGACACGACTTATCGCCACTGGCAGCAGCCACTGGTAACAGGATTAGCAGAGCGAGGTATGTAGGCGGTGCTACAGAGTTCTTGAAGTGGTGGCCTAACTACGGCTACACTAGAAGG
ACAGTATTTGGTATCTGCGCTCTGCTGAAGCCAGTTACCTTCGGAAAAAGAGTTGGTAGCTCTTGATCCGGCAAACAAACCACCGCTGGTAGCGGTGGTTTTTTTGTTTGCAAGCAGCAGATTACGC
GCAGAAAAAAAGGATCTCAAGAAGATCCTTTGATCTTTTCTACGGGGTCTGACGCTCAGTGGAACGAAAACTCACGTTAAGGGATTTTGGTCATGAGATTATCAAAAAGGATCTTCACCTAGATCCT
TTTAAATTAAAAATGAAGTTTTAAATCAATCTAAAGTATATATGAGTAAACTTGGTCTGACAGTTACCAATGCTTAATCAGTGAGGCACCTATCTCAGCGATCTGTCTATTTCGTTCATCCATAGTT
GCCTGACTCCCCGTCGTGTAGATAACTACGATACGGGAGGGCTTACCATCTGGCCCCAGTGCTGCAATGATACCGCGAGACCCACGCTCACCGGCTCCAGATTTATCAGCAATAAACCAGCCAGCCG
GAAGGGCCGAGCGCAGAAGTGGTCCTGCAACTTTATCCGCCTCCATCCAGTCTATTAATTGTTGCCGGGAAGCTAGAGTAAGTAGTTCGCCAGTTAATAGTTTGCGCAACGTTGTTGCCATTGCTAC
AGGCATCGTGGTGTCACGCTCGTCGTTTGGTATGGCTTCATTCAGCTCCGGTTCCCAACGATCAAGGCGAGTTACATGATCCCCCATGTTGTGCAAAAAAGCGGTTAGCTCCTTCGGTCCTCCGATC
GTTGTCAGAAGTAAGTTGGCCGCAGTGTTATCACTCATGGTTATGGCAGCACTGCATAATTCTCTTACTGTCATGCCATCCGTAAGATGCTTTTCTGTGACTGGTGAGTACTCAACCAAGTCATTCT
GAGAATAGTGTATGCGGCGACCGAGTTGCTCTTGCCCGGCGTCAATACGGGATAATACCGCGCCACATAGCAGAACTTTAAAAGTGCTCATCATTGGAAAACGTTCTTCGGGGCGAAAACTCTCAAG
GATCTTACCGCTGTTGAGATCCAGTTCGATGTAACCCACTCGTGCACCCAACTGATCTTCAGCATCTTTTACTTTCACCAGCGTTTCTGGGTGAGCAAAAACAGGAAGGCAAAATGCCGCAAAAAAG
GGAATAAGGGCGACACGGAAATGTTGAATACTCATACTCTTCCTTTTTCAATATTATTGAAGCATTTATCAGGGTTATTGTCTCATGAGCGGATACATATTTGAATGTATTTAGAAAAATAAACAAA
TAGGGGTTCCGCGCACATTTCCCCGAAAAGTGCCACCTGACGCGCCCTGTAGCGGGATCCATTTTATTTAATATACTAAATAATAAAAAAGTTAAAAAATGATCATTGGATAAATTTTTTATAATTA
TAAATAAAGATAATAATTTTTTTTTTAACAAAACTAAAAATAAAAATAATAAAATAATTGTTAAAATAGGTTTTTTTTTTTTTTTTTTTTTTTTAATAAATGGTATTTATTAATTTATTTGTTGTGT
GTGTTTTTTTTTTTATAATATTTTTTTTTTTTAGCATTGAATTAAGAAGAAATCAAATTGATTCTAGTTTAATTTCGGGTATATTTGAGTGGAATGAGTTCTTCAATCGTAGTTTTGACTAACTTGCC
ATTCATTTCTATTAACACAAAACAATCTGGTGCATAGTCTGAAATCAACTCCCTACACATACCACAAGGACTTACCACTCGAATACTTCTATCTACTTCGTCAGAATAAGGGTGTCTAACAGCTACA
ATCGTGTCAAAATCCTTTTGTCCATTCGAAACTGCACTACCAATCGCAATGGCTTCTGCACAAACAGTTACTCGTCCTATATACGCTTCAATATGTACTGCCGAAATGATTTCTCCTGTTTTCGTAC
GAATTGCCGCTCCCACATGATGTTTATTATCCTCATAAAGCATTGTAATCTTCTCTGTCGCTACTTCTACTAATTCTAGATCCTGTTGAGAAATGTTAAATTGATCCATTTTGGATCTTTTATATTA
TATTTATTTATTGATTATTTTTTTGAATTAATTAAAAAAAAAAAAAAATTTCATTTTATAATCTCAGAAACCTCAAAAAAAAAAAAATAAAAAATAAAAAATATAAAAAAATAAAAATAAAATCCCAA
TTTTAAAGCGAAAAACCACCCATGGTTTGAAAATTTCAATCAATTTCAAATAACTTTACTTAAAAAAAACCCATTTTTTATTTAAAAAA
```

Supplementary Figure 1 (*continued*)

```
>pTM1599
CTAAATTGTAAGCGTTAATATTTTGTTAAAATTCGCGTTAAATTTTTGTTAAATCAGCTCATTTTTTAACCAATAGGCCGAAATCGGCAAAATCCCTTATAAATCAAAAGAATAGACCGAGATAGGG
TTGAGTGTTGTTCCAGTTTGGAACAAGAGTCCACTATTAAAGAACGTGGACTCCAACGTCAAAGGGCGAAAAACCGTCTATCAGGGCGATGGCCCACTACGTGAACCATCACCCTAATCAAGTTTTT
TGGGGTCGAGGTGCCGTAAAGCACTAAATCGGAACCCTAAAGGGAGCCCCCGATTTAGAGCTTGACGGGGAAAGCCGGCGAACGTGGCGAGAAAGGAAGGGAAGAAAGCGAAAGGAGCGGGCGCTAG
GGCGCTGGCAAGTGTAGCGGTCACGCTGCGCGTAACCACCACACCCGCCGCGCTTAATGCGCCGCTACAGGGCGCGTCCCATTCGCCATTCAGGCTGCGCAACTGTTGGGAAGGGCGATCGGTGCGG
GCCTCTTCGCTATTACGCCAGCTGGCGAAAGGGGGATGTGCTGCAAGGCGATTAAGTTGGGTAACGCCAGGGTTTTCCCAGTCACGACGTTGTAAAACGACGGCCAGTGAGCGCGCGTAATACGACT
CACTATAGGGCGAATTGGGTACCGGGCCCCCCCTCGAGACTAGATAAAAAAAATTTTTATTTATTTTTATTTATTTTGAATTAAATAGATTACAAATTAATTAATCCCATCAAATCTTTAAAAAAAA
AAATGGTTTAAAAAACTTGGGTTGGTTAATTATTATTTGAAAATTTTAAAACCCAAATTAAAAAAAAAAAAATGGGATTCAAAAATTTTTTTTTTTTTTTTTTTTTTTTTTTTTTTTTTTCAGATTGCA
TAAAAAGATTTTTTTTTTTTTTTTTTTCTTATTTCTTAAAACAAATAAATTAAATTAAATAAAAAAATAAAAATCAGATCTATACAACAATAAAATGGATAAAAAAATATTCAATTGGTTTAGATATTG
GTACAAATAGTGTTGGTTGGGCAGTTATTACAGATGAATATAAAGTTCCATCAAAAAAATTTAAAGTTTTAGGTAATACAGACCGGCATTCCATCAAGAAAAATCTGATCGGAGCTCTCCTCTTTGA
TTCAGGGGAGACCGCTGAAGCAACCCGCCTCAAGCGGACTGCTAGACGGCGGTACACCAGGAGGAAGAACCGGATTTGTTACCTTCAAGAGATATTCTCCAACGAAATGGCAAAGGTCGACGACAGC
TTCTTCCATAGGCTGGAAGAATCATTCCTCGTGGAAGAGGATAAGAAGCATGAACGGCATCCCATCTTCGGTAATATCGTCGACGAGGTGGCCTATCACGAGAAATACCCAACCATCTACCATCTTC
GCAAAAAGCTGGTGGACTCAACCGACAAGGCAGACCTCCGGCTTATCTACCTGGCCCTGGCCCACATGATCAAGTTCAGAGGCCACTTCCTGATCGAGGGCGACCTCAATCCTGACAATAGCGATGT
GGATAAACTGTTCATCCAGCTGGTGCAGACTTACAACCAGCTCTTTGAAGAGAACCCCATCAATGCAAGCGGAGTCGATGCCAAGGCCATTCTGTCAGCCCGGCTGTCAAAGAGCCGCAGACTTGAG
AATCTTATCGCTCAGCTGCCGGGTGAAAAGAAAAATGGACTGTTCGGGAACCTGATTGCTCTTTCACTTGGGCTGACTCCCAATTTCAAGTCTAATTTCGACCTGGCAGAGGATGCCAAGCTGCAAC
TGTCCAAGGACACCTATGATGACGATCTCGACAACCTCCTGGCCCAGATCGGTGACCAATACGCCGACCTTTTCCTTGCTGCTAAGAATCTTTCTGACGCCATCCTGCTGTCTGACATTCTCCGCGT
GAACACTGAAATCACCAAGGCCCCTCTTTCAGCTTCAATGATTAAGCGGTATGATGAGCACCACCAGGACCTGACCCTGCTTAAGGCACTCGTCCGGCAGCAGCTTCCGGAGAAGTACAAGGAAATC
TTCTTTGACCAGTCAAAGAATGGATACGCCGGCTACATCGACGGAGGTGCCTCCCAAGAGGAATTTTATAAGTTTATCAAACCTATCCTTGAGAAGATGGACGGCACCGAAGAGCTCCTCGTGAAAC
TGAATCGGGAGGATCTGCTGCGGAAGCAGCGCACTTTCGACAATGGGAGCATTCCCCACCAGATCCATCTTGGGGAGCTTCACGCCATCCTTCGGCGCCAAGAGGACTTCTACCCCTTTCTTAAGGA
CAACAGGGAGAAGATTGAGAAAATTCTCACTTTCCGCATCCCCTACTACGTGGGACCCCTCGCCAGAGGAAATAGCCGGTTTGCTTGGATGACCAGAAAGTCAGAAGAAACTATCACTCCCTGGAAC
TTCGAAGAGGTGGTGGACAAGGGAGCCAGCGCTCAGTCATTCATCGAACGGATGACTAACTTCGATAAGAACCTCCCCAATGAGAAGGTCCTGCCGAAACATTCCCTGCTCTACGAGTACTTTACCG
TGTACAACGAGCTGACCAAGGTGAAATATGTCACCGAAGGGATGAGGAAGCCCGCATTCCTGTCAGGCGAACAAAAGAAGGCAATTGTGGACCTTCTGTTCAAGACCAATAGAAAGGTGACCGTGAA
GCAGCTGAAGGAGGACTATTTCAAGAAAATTGAATGCTTCGACTCTGTGGAGATTAGCGGGGTCGAAGATCGGTTCAACGCAAGCCTGGGTACCTACCATGATCTGCTTAAGATCATCAAGGACAAG
GATTTTCTGGACAATGAGGAGAACGAGGACATCCTTGAGGACATTGTCCTGACTCTCACTCTGTTCGAGGACCGGGAAATGATCGAGGAGAGGCTTAAGACCTACGCCCATCTGTTCGACGATAAAG
TGATGAAGCAACTTAAACGGAGAAGATATACCGGATGGGGACGCCTTAGCCGCAAACTCATCAACGGAATCCGGGACAAACAGAGCGGAAAGACCATTCTTGATTTCCTTAAGAGCGACGGATTCGC
TAATCGCAACTTCATGCAACTTATCCATGATGATTCCCTGACCTTTAAGGAGGACATCCAGAAGGCCCAAGTGTCTGGACAAGGTGACTCACTGCACGAGCATATCGCAAATCTGGCTGGTTCACCC
GCTATTAAGAAGGGTATTCTCCAGACCGTGAAAGTCGTGGACGAGCTGGTCAAGGTGATGGGTCGCCATAAACCAGAGAACATTGTCATCGAGATGGCCAGGGAAAACCAGACTACCCAGAAGGGAC
AGAAGAACAGCAGGGAGCGGATGAAAAGAATTGAGGAAGGGATTAAGGAGCTCGGGTCACAGATCCTTAAAGAGCACCCGGTGGAAAACACCCAGCTTCAGAATGAGAAGCTCTATCTGTACTACCT
TCAAAATGGACGCGATATGTATGTGGACCAAGAGCTTGATATCAACAGGCTCTCAGACTACGACGTGGACCATATCGTCCCTCAGAGCTTCCTCAAAGACGACTCAATTGACAATAAGGTGCTGACT
CGCTCAGACAAGAACCGGGGAAAGTCAGATAACGTGCCCTCAGAGGAAGTCGTGAAAAAGATGAAGAACTATTGGCGCCAGCTTCTGAACGCAAAGCTGATCACTCAGCGGAAGTTCGACAATCTCA
CTAAGGCTGAGAGGGGCGGACTGAGCGAACTGGACAAAGCAGGATTCATTAAACGGCAACTTGTGGAGACTCGGCAGATTACTAAACATGTCGCCCAAATCCTTGACTCACGCATGAATACCAAGTA
CGACGAAAACGACAAACTTATCCGCGAGGTGAAGGTGATTACCCTGAAGTCCAAGCTGGTCAGCGATTTCAGAAAGGACTTTCAATTCTACAAAGTGCGGGAGATCAATAACTATCATCATGCTCAT
GACGCATATCTGAATGCCGTGGTGGGAACCGCCCTGATCAAGAAGTACCCAAAGCTGGAAAGCGAGTTCGTGTACGGAGACTACAAGGTCTACGACGTGCGCAAGATGATTGCCAAATCTGAGCAGG
AGATCGGAAAGGCCACCGCAAAGTACTTCTTCTACAGCAACATCATGAATTTCTTCAAGACCGAAATCACCCTTGCAAACGGTGAGATCCGGAAGAGGCCGCTCATCGAGACTAATGGGGAGACTGG
CGAAATCGTGTGGGACAAGGGCAGAGATTTCGCTACCGTGCGCAAAGTGCTTTCTATGCCTCAAGTGAACATCGTGAAGAAAACCGAGGTGCAAACCGGAGGCTTTTCTAAGGAATCAATCCTCCCC
AAGCGCAACTCCGACAAGCTCATTGCAAGGAAGAAGGATTGGGACCCTAAGAAGTACGGCGGATTCGATTCACCAACTGTGGCTTATTCTGTCCTGGTCGTGGCTAAGGTGGAAAAAGGAAAGTCTA
AGAAGCTCAAGAGCGTGAAGGAACTGCTGGGTATCACCATTATGGAGCGCAGCTCCTTCGAGAAGAACCCAATTGACTTTCTCGAAGCCAAAGGTTACAAGGAAGTCAAGAAGGACCTTATCATCAA
GCTCCCAAAGTATAGCCTGTTCGAACTGGAGAATGGGCGGAAGCGGATGCTCGCCTCCGCTGGCGAACTTCAGAAGGGTAATGAGCTGGCTCTCCCCTCCAAGTACGTGAATTTCCTCTACCTTGCA
AGCCATTACGAGAAGCTGAAGGGGAGCCCCGAGGACAACGAGCAAAAGCAACTGTTTGTGGAGCAGCATAAGCATTATCTGGACGAGATCATTGAGCAGATTTCCGAGTTTTCTAAACGCGTCATTC
TCGCTGATGCCAACCTCGATAAAGTCCTTAGCGCATACAATAAGCACAGAGACAAACCAATTCGGGAGCAGGCTGAGAATATCATCCACCTGTTCACCCTCACCAATCTTGGTGCCCCTGCCGCATT
CAAGTACTTCGACACCACCATCGACCGGAAACGCTATACCTCCACCAAAGAAGTGCTGGACGCCACCCTCATCCACCAGAGCATCACCGGACTTTACGAAACTCGGATTGACCTCTCACAGCTCGGA
GGGGATGAGGGAGCTCCCAAGAAAAAGCGCAAGGTAGGTAGTTCCATCGCTAGTTAAATAAATAAATTATTTAATAAATAATAAAAAAACAAATTGTTGTAATAATCTAATATTTTCTTTTTTTTTT
AATTTTTTTTTTTTTAAATCTTAATAATTATTAAGTTATTTTAATTTTTTTTTTTTTTTTTTTTTTTTTTTTTTTTTTTTTTCTATCAAAAAAATCAAATATATTTAAAAAATTTATTATTTACAGATACA
TTTTGAATGGTGAAGATAAATATATGCATTAGATGTAAAACAGCCAAAGAGTATGAAAATCAAAAAGATAAAGCTTAAAAAAAGCACCGACTCGGTGCCACTTTTTCAAGTTGATAACGGACTAGCC
TTATTTAAACTTGCTATGCTGTTTCCAGCATAGCTCTTAAACCCGTCTTCCCGAAGACCCTGCTCGATGCAGGGATCGAACCTGCGACCGTGGCGTTATTAGCACCACGCTCTGCCGACTGAGCTAA
TCGAGCCTCGAGTTTTTTAAATAAAAAAATGGGTTTTTTTTAAGTAAAGTTATTTGAAATTGATTGAAATTTTCAAACCATGGGTGGTTTTTCGCTTTAAAATTGGGATTTTATTTTTATTTTTTTAT
ATTTTTTATTTTTTATTTTTTTTTTTTTGAGGTTTCTGAGATTATAAAATGAAATTTTTTTTTTTTTTTTAATTAATTCAAAAAATAATCAAATAAATAAATATAATATAAAAGATCCAAAATGGATG
GTGAAGATGTTCAAGCTGGATCGTTTCGCATGATTGAACAAGATGGATTGCACGCAGGTTCTCCGGCCGCTTGGGTGGAGAGGCTATTCGGCTATGACTGGGCACAACAGACAATCGGCTGCTCTGA
TGCCGCCGTGTTCCGGCTGTCAGCGCAGGGGCGCCCGGTTCTTTTTGTCAAGACCGACCTGTCCGGTGCCCTGAATGAACTGCAGGACGAGGCAGCGCGGCTATCGTGGCTGGCCACGACGGGCGTT
CCTTGCGCAGCTGTGCTCGACGTTGTCACTGAAGCGGGAAGGGACTGGCTGCTATTGGGCGAAGTGCCGGGGCAGGATCTCCTGTCATCTCACCTTGCTCCTGCCGAGAAAGTATCCATCATGGCTG
ATGCAATGCGGCGGCTGCATACGCTTGATCCGGCTACCTGCCCATTCGACCACCAAGCGAAACATCGCATCGAGCGAGCACGTACTCGGATGGAAGCCGGTCTTGTCGATCAGGATGATCTGGACGA
AGAGCATCAGGGGCTCGCGCCAGCCGAACTGTTCGCCAGGCTCAAGGCGCGCATGCCCGACGGCGAGGATCTCGTCGTGACCCATGGCGATGCCTGCTTGCCGAATATCATGGTGGAAAATGGCCGC
TTTTCTGGATTCATCGACTGTGGACGGCTGGGTGTGGCGGACCGCTATCAGGACATAGCGTTGGCTACCCGTGATATTGCTGAAGAGCTTGGCGGCGAATGGGCTGACCGCTTCCTCGTGCTTTACG
GTATCGCCGCTCCCGATTCGCAGCGCATCGCCTTCTATCGCCTTCTTGACGAGTTCTTCTGAACTAGAATCAATTTGATTTCTTCTTAATTCAATGCTAAAAAAAAAAATATTATAAAAAAAAAAAC
ACACACAACAAATAAATTAATAAATACCATTTATTAAAAAAAAAAAAAAAAAAAAAAAACCTATTTTAACAATTATTTTATTATTTTTATTTTTAGTTTTGTTAAAAAAAAAATTATTATCTTTATT
TATAATTATAAAAAATTTATCCAATGATCATTTTTTAACTTTTTTATTATTTAGTATATTAAATAAAATGGATCCACTAGTTCTAGAGCGGCCGCCACCGCGGTGGAGCTCCAGCTTTTGTTCCCTT
TAGTGAGGGTTAATTGCGCGCTTGGCGTAATCATGGTCATAGCTGTTTCCTGTGTGAAATTGTTATCCGCTCACAATTCCACACAACATACGAGCCGGAAGCATAAAGTGTAAAGCCTGGGGTGCCT
AATGAGTGAGCTAACTCACATTAATTGCGTTGCGCTCACTGCCCGCTTTCCAGTCGGGAAACCTGTCGTGCCAGCTGCATTAATGAATCGGCCAACGCGCGGGGAGAGGCGGTTTGCGTATTGGGCG
CTCTTCCGCTTCCTCGCTCACTGACTCGCTGCGCTCGGTCGTTCGGCTGCGGCGAGCGGTATCAGCTCACTCAAAGGCGGTAATACGGTTATCCACAGAATCAGGGGATAACGCAGGAAAGAACATG
TGAGCAAAAGGCCAGCAAAAGGCCAGGAACCGTAAAAAGGCCGCGTTGCTGGCGTTTTTCCATAGGCTCCGCCCCCCTGACGAGCATCACAAAAATCGACGCTCAAGTCAGAGGTGGCGAAACCCGA
CAGGACTATAAAGATACCAGGCGTTTCCCCCTGGAAGCTCCCTCGTGCGCTCTCCTGTTCCGACCCTGCCGCTTACCGGATACCTGTCCGCCTTTCTCCCTTCGGGAAGCGTGGCGCTTTCTCATAG
CTCACGCTGTAGGTATCTCAGTTCGGTGTAGGTCGTTCGCTCCAAGCTGGGCTGTGTGCACGAACCCCCCGTTCAGCCCGACCGCTGCGCCTTATCCGGTAACTATCGTCTTGAGTCCAACCCGGTA
AGACACGACTTATCGCCACTGGCAGCAGCCACTGGTAACAGGATTAGCAGAGCGAGGTATGTAGGCGGTGCTACAGAGTTCTTGAAGTGGTGGCCTAACTACGGCTACACTAGAAGGACAGTATTTG
GTATCTGCGCTCTGCTGAAGCCAGTTACCTTCGGAAAAAGAGTTGGTAGCTCTTGATCCGGCAAACAAACCACCGCTGGTAGCGGTGGTTTTTTTGTTTGCAAGCAGCAGATTACGCGCAGAAAAAA
AGGATCTCAAGAAGATCCTTTGATCTTTTCTACGGGGTCTGACGCTCAGTGGAACGAAAACTCACGTTAAGGGATTTTGGTCATGAGATTATCAAAAAGGATCTTCACCTAGATCCTTTTAAATTAA
AAATGAAGTTTTAAATCAATCTAAAGTATATATGAGTAAACTTGGTCTGACAGTTACCAATGCTTAATCAGTGAGGCACCTATCTCAGCGATCTGTCTATTTCGTTCATCCATAGTTGCCTGACTCC
CCGTCGTGTAGATAACTACGATACGGGAGGGCTTACCATCTGGCCCCAGTGCTGCAATGATACCGCGAGACCCACGCTCACCGGCTCCAGATTTATCAGCAATAAACCAGCCAGCCGGAAGGGCCGA
GCGCAGAAGTGGTCCTGCAACTTTATCCGCCTCCATCCAGTCTATTAATTGTTGCCGGGAAGCTAGAGTAAGTAGTTCGCCAGTTAATAGTTTGCGCAACGTTGTTGCCATTGCTACAGGCATCGTG
GTGTCACGCTCGTCGTTTGGTATGGCTTCATTCAGCTCCGGTTCCCAACGATCAAGGCGAGTTACATGATCCCCCATGTTGTGCAAAAAAGCGGTTAGCTCCTTCGGTCCTCCGATCGTTGTCAGAA
GTAAGTTGGCCGCAGTGTTATCACTCATGGTTATGGCAGCACTGCATAATTCTCTTACTGTCATGCCATCCGTAAGATGCTTTTCTGTGACTGGTGAGTACTCAACCAAGTCATTCTGAGAATAGTG
TATGCGGCGACCGAGTTGCTCTTGCCCGGCGTCAATACGGGATAATACCGCGCCACATAGCAGAACTTTAAAAGTGCTCATCATTGGAAAACGTTCTTCGGGGCGAAAACTCTCAAGGATCTTACCG
CTGTTGAGATCCAGTTCGATGTAACCCACTCGTGCACCCAACTGATCTTCAGCATCTTTTACTTTCACCAGCGTTTCTGGGTGAGCAAAAACAGGAAGGCAAAATGCCGCAAAAAAGGGAATAAGGG
CGACACGGAAATGTTGAATACTCATACTCTTCCTTTTTCAATATTATTGAAGCATTTATCAGGGTTATTGTCTCATGAGCGGATACATATTTGAATGTATTTAGAAAAATAAACAAATAGGGGTTCC
GCGCACATTTCCCCGAAAAGTGCCAC
```

Supplementary Figure 1 (*continued*)

```
>pTM1668
CTAAATTGTAAGCGTTAATATTTTGTTAAAATTCGCGTTAAATTTTTGTTAAATCAGCTCATTTTTTAACCAATAGGCCGAAATCGGCAAAATCCCTTATAAATCAAAAGAATAGACCGAGATAGGG
TTGAGTGTTGTTCCAGTTTGGAACAAGAGTCCACTATTAAAGAACGTGGACTCCAACGTCAAAGGGCGAAAAACCGTCTATCAGGGCGATGGCCCACTACGTGAACCATCACCCTAATCAAGTTTTT
TGGGGTCGAGGTGCCGTAAAGCACTAAATCGGAACCCTAAAGGGAGCCCCCGATTTAGAGCTTGACGGGGAAAGCCGGCGAACGTGGCGAGAAAGGAAGGGAAGAAAGCGAAAGGAGCGGGCGCTAG
GGCGCTGGCAAGTGTAGCGGTCACGCTGCGCGTAACCACCACACCCGCCGCGCTTAATGCGCCGCTACAGGGCGCGTCCCATTCGCCATTCAGGCTGCGCAACTGTTGGGAAGGGCGATCGGTGCGG
GCCTCTTCGCTATTACGCCAGCTGGCGAAAGGGGGATGTGCTGCAAGGCGATTAAGTTGGGTAACGCCAGGGTTTTCCCAGTCACGACGTTGTAAAACGACGGCCAGTGAGCGCGCGTAATACGACT
CACTATAGGGCGAATTGGGTACCGGGCCCCCCCTCGAGACTAGATAAAAAAAATTTTTATTTATTTTTATTTATTTTGAATTAAATAGATTACAAATTAATTAATCCCATCAAATCTTTAAAAAAAA
AAATGGTTTAAAAAACTTGGGTTGGTTAATTATTATTTGAAAATTTTAAAACCCAAATTAAAAAAAAAAAAATGGGATTCAAAAATTTTTTTTTTTTTTTTTTTTTTTTTTTTTTTTTTTCAGATTGCA
TAAAAAGATTTTTTTTTTTTTTTTTTTCTTATTTCTTAAAACAAATAAATTAAATTAAATAAAAAAATAAAAATCAGATCTATACAACAATAAAATGGATAAAAAAATACTCTATAGGACTTGATATAG
GTACAAATAGTGTTGGATGGGCCGTAATTACAGATGAATACAAAGTACCTTCTAAAAAGTTTAAAGTTCTTGGAAATACTGATAGACACTCAATTAAGAAGAATTTGATAGGTGCCTTGTTATTCGA
TTCTGGAGAAACAGCTGAGAGAACACGTTTGAAGAGAACCGCTAGACGTCGTTACACTCGTCGTAAAAATCGTATTTGTTATTTACAAGAAATATTCAGTAATGAGATGGCAAAAGTAGATGATAGT
TTTTTCCATCGTTTAGAGGAATCATTCCTTGTAGAAGAGGATAAGAAGCATGAGCGTCATCCTATATTTGGAAATATAGTAGATGAGGTAGCCTACCACGAAAAGTATCCAACTATATATCACTTGA
GAAAGAAGTTAGTAGATTCAACTGATAAAGCCGATCTTCGTTTGATATATCTTGCCTTAGCTCATATGATTAAGTTCCGTGGACATTTCTTAATCGAAGGTGATTTGAATCCTGATAATTCAGATGT
AGATAAGTTGTTTATCCAACTTGTTCAAACATACAATCAATTATTTGAAGAGAATCCTATCAATGCCTCAGGAGTAGATGCAAAAGCTATCTTATCAGCACGTTTGAGTAAATCACGTCGTTTGGAA
AATCTTATTGCACAATTACCTGGTGAGAAGAAAAATGGTCTTTTCGGAAATCTTATCGCCTTATCTCTTGGACTTACACCTAATTTTAAGAGTAATTTTGATTTGGCTGAGGATGCCAAGTTGCAAT
TAAGTAAGGATACCTACGATGATGATCTTGATAATTTATTGGCCCAAATAGGAGATCAATATGCAGATTTGTTCCTTGCTGCAAAGAATTTATCTGATGCCATCCTTTTATCAGATATTTTGAGAGT
TAATACAGAGATTACTAAGGCCCCACTTTCTGCCAGTATGATTAAAAGATATGATGAGCATCACCAAGATTTAACTCTTTTGAAAGCTCTTGTTCGTCAACAATTACCAGAGAAGTACAAGGAGATA
TTCTTTGATCAAAGTAAGAATGGTTACGCTGGATACATTGATGGAGGTGCTTCTCAAGAAGAGTTCTACAAGTTTATTAAGCCTATACTTGAGAAAATGGATGGTACAGAGGAATTACTTGTTAAAT
TAAATAGAGAAGATTTACTTAGAAAGCAACGTACATTTGATAATGGATCTATTCCTCATCAAATTCACCTTGGAGAGTTACATGCCATATTGAGAAGACAAGAGGATTTCTATCCTTTCCTTAAAGA
TAATAGAGAGAAGATTGAGAAGATTTTAACCTTTCGTATTCCTTATTATGTAGGACCTTTGGCTCGTGGAAATAGTAGATTTGCATGGATGACAAGAAAATCAGAGGAAACTATCACCCCTTGGAAT
TTTGAAGAAGTTGTAGATAAAGGTGCATCTGCTCAAAGTTTTATCGAAAGAATGACTAATTTCGATAAGAATTTACCTAATGAGAAGGTACTTCCAAAACATTCATTATTATACGAGTATTTTACAG
TTTATAATGAGCTTACTAAGGTAAAGTATGTAACCGAAGGTATGAGAAAGCCTGCATTTCTTAGTGGAGAACAAAAAAAAGCTATTGTAGATTTGTTATTCAAGACCAATAGAAAAGTTACAGTAAA
GCAATTAAAGGAGGATTACTTCAAGAAGATTGAGTGTTTCGATAGTGTTGAGATTTCAGGAGTTGAGGATAGATTCAATGCTTCATTGGGTACCTACCACGATTTGTTGAAGATCATCAAGGATAAA
GATTTTCTTGATAATGAAGAAAATGAAGATATATTAGAAGATATAGTTTTAACTTTAACATTGTTCGAAGATCGTGAAATGATAGAGGAACGTTTGAAAACTTATGCCCATCTTTTCGATGATAAAG
TTATGAAACAATTGAAAAGAAGACGTTATACTGGTTGGGGACGTTTAAGTCGTAAGTTAATAAATGGTATTCGTGATAAGCAATCAGGTAAGACCATTTTAGATTTTTTGAAATCAGATGGATTTGC
TAATAGAAATTTCATGCAATTAATACACGATGATTCTTTAACTTTTAAGGAGGATATACAAAAAGCTCAAGTTTCTGGACAAGGAGATTCTTTACATGAACACATAGCAAATCTTGCTGGTTCACCT
GCAATAAAGAAAGGAATCTTGCAAACTGTTAAAGTTGTTGATGAATTGGTTAAGGTAATGGGTCGTCATAAACCTGAGAATATCGTTATCGAAATGGCCCGTGAAAATCAAACTACCCAAAAGGGAC
AAAAGAATAGTCGTGAACGTATGAAGCGTATCGAGGAAGGTATAAAGGAATTGGGATCTCAAATATTAAAGGAACATCCTGTAGAAAATACACAATTGCAAAATGAAAAGTTATATTTGTATTACTT
ACAAAATGGTCGTGATATGTATGTTGATCAAGAATTGGATATAAATAGATTATCTGATTATGATGTAGATCATATTGTTCCACAAAGTTTTTTAAAAGATGATTCTATTGATAATAAGGTTCTTACT
CGTTCTGATAAAAATCGTGGAAAGTCTGATAATGTTCCAAGTGAAGAAGTTGTAAAAAAGATGAAGAATTACTGGCGTCAATTATTAAATGCCAAATTGATAACACAACGTAAGTTTGATAATCTTA
CTAAAGCAGAGAGAGGTGGACTTTCAGAATTGGATAAGGCCGGATTTATAAAACGTCAATTAGTAGAGACCCGTCAAATTACTAAGCATGTAGCTCAAATACTTGATAGTAGAATGAATACCAAGTA
TGATGAAAATGATAAGTTAATTCGTGAAGTAAAGGTTATAACCCTTAAGAGTAAGTTAGTAAGTGATTTTCGTAAAGATTTCCAATTTTATAAAGTAAGAGAGATAAATAATTATCACCACGCCCAC
GATGCCTACTTAAATGCTGTAGTAGGTACTGCTCTTATAAAAAAATATCCTAAGTTGGAAAGTGAATTTGTATATGGAGATTATAAAGTATATGATGTTCGTAAAATGATCGCAAAGAGTGAGCAAG
AGATTGGTAAGGCTACAGCAAAGTACTTCTTCTATTCAAATATAATGAATTTCTTCAAGACAGAGATTACACTTGCTAATGGTGAAATACGTAAGCGTCCTTTAATTGAGACTAATGGTGAAACTGG
TGAAATAGTATGGGATAAGGGAAGAGATTTCGCTACTGTTCGTAAAGTACTTAGTATGCCACAAGTAAATATAGTTAAAAAAACTGAGGTACAAACTGGAGGTTTCTCTAAGGAGTCAATCCGTCCA
AAGCGTAATAGTGATAAATTGATAGCACGTAAAAAAGATTGGGACCCAAAGAAATATGGTGGTTTTTTATGGCCTACCGTTGCATATTCAGTTTTAGTAGTAGCAAAGGTTGAGAAAGGTAAATCAA
AAAAGTTGAAATCAGTTAAGGAGTTACTTGGAATTACAATAATGGAAAGATCATCTTTTGAGAAAAATCCTATTGATTTTTTGGAGGCAAAAGGATATAAAGAGGTTAAAAAGGATTTAATAATAAA
ATTGCCTAAGTACAGTCTTTTCGAATTAGAAAATGGTAGAAAACGTATGTTAGCCAGTGCTAAGCAATTGCAAAAGGGAAATGAACTTGCTCTTCCTTCAAAATACGTTAATTTTCTTTACCTTGCA
TCTCATTATGAGAAGTTAAAGGGTTCTCCAGAAGATAATGAGCAAAAACAATTGTTTGTAGAACAACACAAGCATTACTTGGATGAGATTATCGAACAAATATCAGAATTTTCAAAGAGAGTAATTT
TGGCAGATGCAAATCTTGATAAAGTTTTATCAGCATACAATAAGCATAGAGATAAGCCTATTCGTGAACAAGCCGAAAATATAATCCACTTGTTTACCTTAACTAGATTAGGTGCACCTAGAGCTTT
CAAGTATTTCGATACCACAATTGATCCAAAGCAATATCGTAGTACCAAAGAAGTTTTAGATGCTACTCTTATTCACCAAAGTATTACAGGTTTATATGAGACCCGTATTGATCTTTCTCAACTTGGT
GGTGATGAAGGTGCCCCTAAAAAGAAAAGAAAAGTTGGTTCATCATAAACTAGTTAAATAAATAAATTATTTAATAAATAATAAAAAAACAAATTGTTGTAATAATCTAATATTTTCTTTTTTTTTT
AATTTTTTTTTTTTTAAATCTTAATAATTATTAAGTTATTTTAATTTTTTTTTTTTTTTTTTTTTTTTTTTTTTTTTTTTTCTATCAAAAAAATCAAATATATTTAAAAAATTTATTATTTACAGATACA
TTTTGAATGGTGAAGATAAATATATGCATTAGATGTAAAACAGCCAAAGAGTATGAAAATCAAAAAGATAAAGCTTAAAAAAAGCACCGACTCGGTGCCACTTTTTCAAGTTGATAACGGACTAGCC
TTATTTAAACTTGCTATGCTGTTTCCAGCATAGCTCTTAAACCGAGACGCCCGTCTCCTGCTCGATGCAGGGATCGAACCTGCGACCGTGGCGTTATTAGCACCACGCTCTGCCGACTGAGCTAATC
GAGCCTCGAGTTTTTTAAATAAAAAAATGGGTTTTTTTTAAGTAAAGTTATTTGAAATTGATTGAAATTTTCAAACCATGGGTGGTTTTTCGCTTTAAAATTGGGATTTTATTTTTATTTTTTTATAT
TTTTTATTTTTTATTTTTTTTTTTTTTGAGGTTTCTGAGATTATAAAATGAAATTTTTTTTTTTTTTTTAATTAATTCAAAAAATAATCAAATAAATAAATATAATATAAAAGATCCAAAATGGATGGT
GAAGATGTTCAAGCTGGATCGTTTCGCATGATTGAACAAGATGGATTGCACGCAGGTTCTCCGGCCGCTTGGGTGGAGAGGCTATTCGGCTATGACTGGGCACAACAGACAATCGGCTGCTCTGATG
CCGCCGTGTTCCGGCTGTCAGCGCAGGGGCGCCCGGTTCTTTTTGTCAAGACCGACCTGTCCGGTGCCCTGAATGAACTGCAGGACGAGGCAGCGCGGCTATCGTGGCTGGCCACGACGGGCGTTCC
TTGCGCAGCTGTGCTCGACGTTGTCACTGAAGCGGGAAGGGACTGGCTGCTATTGGGCGAAGTGCCGGGGCAGGATCTCCTGTCATCTCACCTTGCTCCTGCCGAGAAAGTATCCATCATGGCTGAT
GCAATGCGGCGGCTGCATACGCTTGATCCGGCTACCTGCCCATTCGACCACCAAGCGAAACATCGCATCGAGCGAGCACGTACTCGGATGGAAGCCGGTCTTGTCGATCAGGATGATCTGGACGAAG
AGCATCAGGGGCTCGCGCCAGCCGAACTGTTCGCCAGGCTCAAGGCGCGCATGCCCGACGGCGAGGATCTCGTCGTGACCCATGGCGATGCCTGCTTGCCGAATATCATGGTGGAAAATGGCCGCTT
TTCTGGATTCATCGACTGTGGACGGCTGGGTGTGGCGGACCGCTATCAGGACATAGCGTTGGCTACCCGTGATATTGCTGAAGAGCTTGGCGGCGAATGGGCTGACCGCTTCCTCGTGCTTTACGGT
ATCGCCGCTCCCGATTCGCAGCGCATCGCCTTCTATCGCCTTCTTGACGAGTTCTTCTGAACTAGAATCAATTTGATTTCTTCTTAATTCAATGCTAAAAAAAAAAATATTATAAAAAAAAAAAACAC
ACACAACAAATAAATTAATAAATACCATTTATTAAAAAAAAAAAAAAAAAAAAAAAACCTATTTTAACAATTATTTTATTATTTTTATTTTTAGTTTTGTTAAAAAAAAAATTATTATCTTTATTTA
TAATTATAAAAAATTTATCCAATGATCATTTTTTAACTTTTTTATTATTTAGTATATTAAATAAAATGGATCCACTAGTTCTAGAGCGGCCGCCACCGCGGTGGAGCTCCAGCTTTTGTTCCCTTTA
GTGAGGGTTAATTGCGCGCTTGGCGTAATCATGGTCATAGCTGTTTCCTGTGTGAAATTGTTATCCGCTCACAATTCCACACAACATACGAGCCGGAAGCATAAAGTGTAAAGCCTGGGGTGCCTAA
TGAGTGAGCTAACTCACATTAATTGCGTTGCGCTCACTGCCCGCTTTCCAGTCGGGAAACCTGTCGTGCCAGCTGCATTAATGAATCGGCCAACGCGCGGGGAGAGGCGGTTTGCGTATTGGGCGCT
CTTCCGCTTCCTCGCTCACTGACTCGCTGCGCTCGGTCGTTCGGCTGCGGCGAGCGGTATCAGCTCACTCAAAGGCGGTAATACGGTTATCCACAGAATCAGGGGATAACGCAGGAAAGAACATGTG
AGCAAAAGGCCAGCAAAAGGCCAGGAACCGTAAAAAGGCCGCGTTGCTGGCGTTTTTCCATAGGCTCCGCCCCCCTGACGAGCATCACAAAAATCGACGCTCAAGTCAGAGGTGGCGAAACCCGACA
GGACTATAAAGATACCAGGCGTTTCCCCCTGGAAGCTCCCTCGTGCGCTCTCCTGTTCCGACCCTGCCGCTTACCGGATACCTGTCCGCCTTTCTCCCTTCGGGAAGCGTGGCGCTTTCTCATAGCT
CACGCTGTAGGTATCTCAGTTCGGTGTAGGTCGTTCGCTCCAAGCTGGGCTGTGTGCACGAACCCCCCGTTCAGCCCGACCGCTGCGCCTTATCCGGTAACTATCGTCTTGAGTCCAACCCGGTAAG
ACACGACTTATCGCCACTGGCAGCAGCCACTGGTAACAGGATTAGCAGAGCGAGGTATGTAGGCGGTGCTACAGAGTTCTTGAAGTGGTGGCCTAACTACGGCTACACTAGAAGGACAGTATTTGGT
ATCTGCGCTCTGCTGAAGCCAGTTACCTTCGGAAAAAGAGTTGGTAGCTCTTGATCCGGCAAACAAACCACCGCTGGTAGCGGTGGTTTTTTTGTTTGCAAGCAGCAGATTACGCGCAGAAAAAAAG
GATCTCAAGAAGATCCTTTGATCTTTTCTACGGGGTCTGACGCTCAGTGGAACGAAAACTCACGTTAAGGGATTTTGGTCATGAGATTATCAAAAAGGATCTTCACCTAGATCCTTTTAAATTAAAA
ATGAAGTTTTAAATCAATCTAAAGTATATATGAGTAAACTTGGTCTGACAGTTACCAATGCTTAATCAGTGAGGCACCTATCTCAGCGATCTGTCTATTTCGTTCATCCATAGTTGCCTGACTCCCC
GTCGTGTAGATAACTACGATACGGGAGGGCTTACCATCTGGCCCCAGTGCTGCAATGATACCGCGAGACCCACGCTCACCGGCTCCAGATTTATCAGCAATAAACCAGCCAGCCGGAAGGGCCGAGC
GCAGAAGTGGTCCTGCAACTTTATCCGCCTCCATCCAGTCTATTAATTGTTGCCGGGAAGCTAGAGTAAGTAGTTCGCCAGTTAATAGTTTGCGCAACGTTGTTGCCATTGCTACAGGCATCGTGGT
GTCACGCTCGTCGTTTGGTATGGCTTCATTCAGCTCCGGTTCCCAACGATCAAGGCGAGTTACATGATCCCCCATGTTGTGCAAAAAAGCGGTTAGCTCCTTCGGTCCTCCGATCGTTGTCAGAAGT
AAGTTGGCCGCAGTGTTATCACTCATGGTTATGGCAGCACTGCATAATTCTCTTACTGTCATGCCATCCGTAAGATGCTTTTCTGTGACTGGTGAGTACTCAACCAAGTCATTCTGAGAATAGTGTA
TGCGGCGACCGAGTTGCTCTTGCCCGGCGTCAATACGGGATAATACCGCGCCACATAGCAGAACTTTAAAAGTGCTCATCATTGGAAAACGTTCTTCGGGGCGAAAACTCTCAAGGATCTTACCGCT
GTTGAGATCCAGTTCGATGTAACCCACTCGTGCACCCAACTGATCTTCAGCATCTTTTACTTTCACCAGCGTTTCTGGGTGAGCAAAAACAGGAAGGCAAAATGCCGCAAAAAAGGGAATAAGGGCG
ACACGGAAATGTTGAATACTCATACTCTTCCTTTTTCAATATTATTGAAGCATTTATCAGGGTTATTGTCTCATGAGCGGATACATATTTGAATGTATTTAGAAAAATAAACAAATAGGGGTTCCGC
GCACATTTCCCCGAAAAGTGCCAC
```

Supplementary Figure 1 (*continued*)

```
>pTM1709
GGATCCCCCCTCGAGACTAGATAAAAAAAATTTTTATTTATTTTTATTTATTTTGAATTAAATAGATTACAAATTAATTAATCCCATCAAATCTTTAAAAAAAAAAATGGTTTAAAAAAACTTGGGT
TGGTTAATTATTATTTGAAAATTTTAAAACCCAAATTAAAAAAAAAAAATGGGATTCAAAAAATTTTTTTTTTTTTTTTTTTTTTTTTTTTTTTTTTCAGATTGCATAAAAAGATTTTTTTTTTTTT
TTTTTTCTTATTTCTTAAAACAAATAAATTAAATTAAATAAAAAATAAAAATCAGATCTATACAACAATAAAATGGATAAAAAATATTCAATTGGTTTAGATATTGGTACAAATAGTGTTGGTTGGG
CAGTTATTACAGATGAATATAAAGTTCCATCAAAAAAATTTAAAGTTTTAGGTAATACAGACCGGCATTCCATCAAGAAAAATCTGATCGGAGCTCTCCTCTTTGATTCAGGGGAGACCGCTGAAGC
AACCCGCCTCAAGCGGACTGCTAGACGGCGGTACACCAGGAGGAAGAACCGGATTTGTTACCTTCAAGAGATATTCTCCAACGAAATGGCAAAGGTCGACGACAGCTTCTTCCATAGGCTGGAAGAA
TCATTCCTCGTGGAAGAGGATAAGAAGCATGAACGGCATCCCATCTTCGGTAATATCGTCGACGAGGTGGCCTATCACGAGAAATACCCAACCATCTACCATCTTCGCAAAAAGCTGGTGGACTCAA
CCGACAAGGCAGACCTCCGGCTTATCTACCTGGCCCTGGCCCACATGATCAAGTTCAGAGGCCACTTCCTGATCGAGGGCGACCTCAATCCTGACAATAGCGATGTGGATAAACTGTTCATCCAGCT
GGTGCAGACTTACAACCAGCTCTTTGAAGAGAACCCCATCAATGCAAGCGGAGTCGATGCCAAGGCCATTCTGTCAGCCCGGCTGTCAAAGAGCCGCAGACTTGAGAATCTTATCGCTCAGCTGCCG
GGTGAAAAGAAAAATGGACTGTTCGGGAACCTGATTGCTCTTTCACTTGGGCTGACTCCCAATTTCAAGTCTAATTTCGACCTGGCAGAGGATGCCAAGCTGCAACTGTCCAAGGACACCTATGATG
ACGATCTCGACAACCTCCTGGCCCAGATCGGTGACCAATACGCCGACCTTTTCCTTGCTGCTAAGAATCTTTCTGACGCCATCCTGCTGTCTGACATTCTCCGCGTGAACACTGAAATCACCAAGGC
CCCTCTTTCAGCTTCAATGATTAAGCGGTATGATGAGCACCACCAGGACCTGACCCTGCTTAAGGCACTCGTCCGGCAGCAGCTTCCGGAGAAGTACAAGGAAATCTTCTTTGACCAGTCAAAGAAT
GGATACGCCGGCTACATCGACGGAGGTGCCTCCCAAGAGGAATTTTATAAGTTTATCAAACCTATCCTTGAGAAGATGGACGGCACCGAAGAGCTCCTCGTGAAACTGAATCGGGAGGATCTGCTGC
GGAAGCAGCGCACTTTCGACAATGGGAGCATTCCCCACCAGATCCATCTTGGGGAGCTTCACGCCATCCTTCGGCGCCAAGAGGACTTCTACCCCTTTCTTAAGGACAACAGGGAGAAGATTGAGAA
AATTCTCACTTTCCGCATCCCCTACTACGTGGGACCCCTCGCCAGAGGAAATAGCCGGTTTGCTTGGATGACCAGAAAGTCAGAAGAAACTATCACTCCCTGGAACTTCGAAGAGGTGGTGGACAAG
GGAGCCAGCGCTCAGTCATTCATCGAACGGATGACTAACTTCGATAAGAACCTCCCCAATGAGAAGGTCCTGCCGAAACATTCCCTGCTCTACGAGTACTTTACCGTGTACAACGAGCTGACCAAGG
TGAAATATGTCACCGAAGGGATGAGGAAGCCCGCATTCCTGTCAGGCGAACAAAAGAAGGCAATTGTGGACCTTCTGTTCAAGACCAATAGAAAGGTGACCGTGAAGCAGCTGAAGGAGGACTATTT
CAAGAAAATTGAATGCTTCGACTCTGTGGAGATTAGCGGGGTCGAAGATCGGTTCAACGCAAGCCTGGGTACCTACCATGATCTGCTTAAGATCATCAAGGACAAGGATTTTCTGGACAATGAGGAG
AACGAGGACATCCTTGAGGACATTGTCCTGACTCTCACTCTGTTCGAGGACCGGGAAATGATCGAGGAGAGGCTTAAGACCTACGCCCATCTGTTCGACGATAAAGTGATGAAGCAACTTAAACGGA
GAAGATATACCGGATGGGGACGCCTTAGCCGCAAACTCATCAACGGAATCCGGGACAAACAGAGCGGAAAGACCATTCTTGATTTCCTTAAGAGCGACGGATTCGCTAATCGCAACTTCATGCAACT
TATCCATGATGATTCCCTGACCTTTAAGGAGGACATCCAGAAGGCCCAAGTGTCTGGACAAGGTGACTCACTGCACGAGCATATCGCAAATCTGGCTGGTTCACCCGCTATTAAGAAGGGTATTCTC
CAGACCGTGAAAGTCGTGGACGAGCTGGTCAAGGTGATGGGTCGCCATAAACCAGAGAACATTGTCATCGAGATGGCCAGGGAAAACCAGACTACCCAGAAGGGACAGAAGAACAGCAGGGAGCGGA
TGAAAAGAATTGAGGAAGGGATTAAGGAGCTCGGGTCACAGATCCTTAAAGAGCACCCGGTGGAAAACACCCAGCTTCAGAATGAGAAGCTCTATCTGTACTACCTTCAAAATGGACGCGATATGTA
TGTGGACCAAGAGCTTGATATCAACAGGCTCTCAGACTACGACGTGGACCATATCGTCCCTCAGAGCTTCCTCAAAGACGACTCAATTGACAATAAGGTGCTGACTCGCTCAGACAAGAACCGGGGA
AAGTCAGATAACGTGCCCTCAGAGGAAGTCGTGAAAAAGATGAAGAACTATTGGCGCCAGCTTCTGAACGCAAAGCTGATCACTCAGCGGAAGTTCGACAATCTCACTAAGGCTGAGAGGGGCGGAC
TGAGCGAACTGGACAAAGCAGGATTCATTAAACGGCAACTTGTGGAGACTCGGCAGATTACTAAACATGTCGCCCAAATCCTTGACTCACGCATGAATACCAAGTACGACGAAAACGACAAACTTAT
CCGCGAGGTGAAGGTGATTACCCTGAAGTCCAAGCTGGTCAGCGATTTCAGAAAGGACTTTCAATTCTACAAAGTGCGGGAGATCAATAACTATCATCATGCTCATGACGCATATCTGAATGCCGTG
GTGGGAACCGCCCTGATCAAGAAGTACCCAAAGCTGGAAAGCGAGTTCGTGTACGGAGACTACAAGGTCTACGACGTGCGCAAGATGATTGCCAAATCTGAGCAGGAGATCGGAAAGGCCACCGCAA
AGTACTTCTTCTACAGCAACATCATGAATTTCTTCAAGACCGAAATCACCCTTGCAAACGGTGAGATCCGGAAGAGGCCGCTCATCGAGACTAATGGGGAGACTGGCGAAATCGTGTGGGACAAGGG
CAGAGATTTCGCTACCGTGCGCAAAGTGCTTTCTATGCCTCAAGTGAACATCGTGAAGAAAACCGAGGTGCAAACCGGAGGCTTTTCTAAGGAATCAATCCTCCCCAAGCGCAACTCCGACAAGCTC
ATTGCAAGGAAGAAGGATTGGGACCCTAAGAAGTACGGCGGATTCGATTCACCAACTGTGGCTTATTCTGTCCTGGTCGTGGCTAAGGTGGAAAAAGGAAAGTCTAAGAAGCTCAAGAGCGTGAAGG
AACTGCTGGGTATCACCATTATGGAGCGCAGCTCCTTCGAGAAGAACCCAATTGACTTTCTCGAAGCCAAAGGTTACAAGGAAGTCAAGAAGGACCTTATCATCAAGCTCCCAAAGTATAGCCTGTT
CGAACTGGAGAATGGGCGGAAGCGGATGCTCGCCTCCGCTGGCGAACTTCAGAAGGGTAATGAGCTGGCTCTCCCCTCCAAGTACGTGAATTTCCTCTACCTTGCAAGCCATTACGAGAAGCTGAAG
GGGAGCCCCGAGGACAACGAGCAAAAGCAACTGTTTGTGGAGCAGCATAAGCATTATCTGGACGAGATCATTGAGCAGATTTCCGAGTTTTCTAAACGCGTCATTCTCGCTGATGCCAACCTCGATA
AAGTCCTTAGCGCATACAATAAGCACAGAGACAAACCAATTCGGGAGCAGGCTGAGAATATCATCCACCTGTTCACCCTCACCAATCTTGGTGCCCCTGCCGCATTCAAGTACTTCGACACCACCAT
CGACCGGAAACGCTATACCTCCACCAAAGAAGTGCTGGACGCCACCCTCATCCACCAGAGCATCACCGGACTTTACGAAACTCGGATTGACCTCTCACAGCTCGGAGGGGATGAGGGAGCTCCCAAG
AAAAAGCGCAAGGTAGGTAGTTCCATCGCTAGTtaaATAAATAAATTATTTAATAAATAATAAAAAAACAAATTGTTGTAATAATCTAATATTTTCTTTTTTTTTTAATTTTTTTTTTTTAAATCTT
AATAATTATTAAGTTATTTAATTTTTTTTTTTTTTTTTTTTTTTTTTTTTTTTTTTTTTTTCTATCAAAAAAATCAAATATATTTAAAAAATTTATTATTTACAGATACATTTTGAATGGTGAAGATA
AATATATGCATTAGATGTAAAACAGCCAAAGAGTATGAAAATCAAAAAGATAAAGCTTAAAAAAAGCACCGACTCGGTGCCACTTTTTCAAGTTGATAACGGACTAGCCTTATTTAAACTTGCTATG
CTGTTTCCAGCATAGCTCTTAAACCGAGACGCCCGTCTCCTGCTCGATGCAGGGATCGAACCTGCGACCGTGGCGTTATTAGCACCACGCTCTGCCGACTGAGCTAATCGAGCCTCGAGTTTTTTAA
ATAAAAAATGGGTTTTTTTTAAGTAAAGTTATTTGAAATTGATTGAAATTTTCAAACCATGGGTGGTTTTTCGCTTTAAAATTGGGATTTTATTTTTATTTTTTTATATTTTTTATTTTTTATTTTT
TTTTTTTTGAGGTTTCTGAGATTATAAAATGAAATTTTTTTTTTTTTTTTAATTAATTCAAAAAATAATCAAATAAATAAATATAATATAAAAGATCCAAAATGGATCAATTTAACATTTCTCAACAG
GATCTAGAATTAGTAGAAGTAGCGACAGAGAAGATTACAATGCTTTATGAGGATAATAAACATCATGTGGGAGCGGCAATTCGTACGAAAACAGGAGAAATCATTTCGGCAGTACATATTGAAGCGT
ATATAGGACGAGTAACTGTTTGTGCAGAAGCCATTGCGATTGGTAGTGCAGTTTCGAATGGACAAAAGGATTTTGACACGATTGTAGCTGTTAGACACCCTTATTCTGACGAAGTAGATAGAAGTAT
TCGAGTGGTAAGTCCTTGTGGTATGTGTAGGGAGTTGATTTCAGACTATGCACCAGATTGTTTTGTGTTAATAGAAATGAATGGCAAGTTAGTCAAAACTACGATTGAAGAACTCATTCCACTCAAA
TATACCCGAAATTAAACTAGAATCAATTTGATTTTTTTTTAATTCAATGCTAAAAAAAAAAATATTATAAAAAAAAAAACACCCCCAACAAATAAATTAATAAATACCATTTATTAAAAAAAAAAAA
AAAAAAAAACCTATTTTAACAATTATTTTATTATTTTTATTTTTAGTTTTGTTAAAAAAAAAATTATTATCTTTATTTATAATTATAAAAAATTTATCCAATGATCATTTTTTAACTTTTTTATTAT
TTAGTATATTAAATAAAATGGATCCTATCGATTTCGAAAAAGTAAATAGCAATTATTACAAAATTCAATCCGAATCTACCCAAATAAATTCCAATGAAATTGCCGATTTAAAAAAGTTTATTAAAGA
AGAAGTCAATAAAACTTCTTCCAAAATTGATTTCTTTTTAGTTTCTTCAACAGATGCCCTTTCAAATCCAGAAAATTATTCTCTCTTAGAAGTAAAGTGTATTAATTGTCATTCTTTGTGTCAAGGA
AAAAATTTATATATTTCATGTACAAGAGATGGATGTCAAAACAATATTTGCTATAATTGTTTAGGAATAAACATAAACATATATAATGTTGTTATTAATTCTAAACTTTGCCCTCCATGTTTCAATG
ATTCGGTAATCAACAAGAAGTGTGCCATGTGTAGTAAGAACGGAACTAAATGTAATTTGAACCAAGAATGTAAACTTCATCTTTGTGCACAGTGTTCTAAAAAGTGTCTATACATTCTGAGAGTCAA
AACTAATTAAATAAAATATAAACTTAATTTCTAAATAAACTCATTTAAAAATATTTAAATAATATGAATTTATAACTGTAATTATTGTATTAAAAAATTATATAATTATTTAATGTTAAAAATGTAT
TAAAATAATTATAAAAAAATATAACAAAAATTTTCGTAAAAATAATTTGTAAAAAAGCTATTAAAAATATTATGAAAAAAAAATTAAAAAAATTATTAAATTGTTTTTGTAATTAAGCTATTAAAAT
AATTATAAAAAAAAAATTTTTAAAATTTTAAAAATATTTTTTGTAAAAAAGTATTAAAATAATTATGAAAAAAAAATTTTCTAAAAAATTAAAAAAAAAATTAAAATATATTTTATGTTAAAAACGT
ATTAAAATAACTATTAAAAAAATTATATTTAAAAAAGTATTAACTTTTTTTTAGGTGTGGTTGTGGGGTGGGGTTTAATATATTATAATAAAAAATTATTTTTTGTTCATTTATTATTTTCATTGTA
TATAATGTACTCAACAACGTTATTATTTTTTCTTTTTTTTTTTATTGTATCAAAATCTTCTGTTCTTCAAAATGATCAGATTGAAGTAAAATATTTTCAACTTCTTATTGTTATGTATCAAAAAGAA
AACTGTGTTGAAAAGTCAATGACAGGCGCCGTAATTTATGATGAATGTAATATTCATGGAAGAGTTGAAACAAATAGTACTCATGCGCTTTTTTATGATGACATTGAAACAAATAATTCAAGATGTA
ACAATTTTCGTAATTTAACAAACTTAATTAAACTTAATGAATGTATTAATGACGAGTTTGGAGAGTCTATTCTTTATAAAGAATATAATGAAACTGATGATGGTTATTTGTTTAGAGTGGAAGACAG
CTTTGTTGAAATTACTTCTCTTTCAATGGATTGTACAAAAAATAGTAAAACAATTATTGAAAAATTCAACATTTGTTCAAAATTTGAAAATGTATATCATATTACAAACATTACACAAGAGAAATCC
AATAGATTTACATGTACAGATCCATTGTGCCACTATTGTAAGAATGAAAACATTCAAAACAATCTTGATTTTAAAACAACAAAGTGTACTCCAAAGTATGGTGCATCTGATTCTGAATTTTTATCAA
CAATTTACAATCCAAAGCTCGATGGCTCAAATAACGGTATGGAAAAGTCAGTAACTCAAGAAAAAAACATTTCAAATAATTTAAAAATTAATATATATTTAATTTCTTTTTAATTATTTTTTTAAT
TAAATAAAGTTTTATTATTTTTTAAGAGTAATTATTGCTCTTTTTTCATTTGAAACACCAGAAGCTAAACGTAATTGTTGTTGACTGAAATTTTTTATTTTTTTTGGGGTAATAGGATTTCCTTTTT
TATGAAGATTAATATCTTTGACTCGTGAAACATTCTTTTTAACTTTTGTTTTTTCTGTTGGTTTATCATTTGTTTTTTCACTAATTTCAATACCATCTTGACGTTCATTCATAACTTCATCTTTTTT
TTTTCCTGTTTCTGTATCTTCTTCTATTTTTTTTTCTTTATCTTTTTCTTTATCTTCTTCTTGTTCTTCCTCTTCTTTTTCTTCTTCTGATACTGCAGGTGTTTCTTCTTCTTCTTCTTCCGATATT
GTCGGTTTTTCTACTTCTTCTTCTTGTTCTTCTTCTTCTTCTTCTTCTTCTTCTTCTTCTTCTTCTTCTTCTTCTTCTTCTTCCGGTAATTTATTAATTATATTTCTTTTTTTATATGAATTACGTTTGG
TTTGTGCAGTAATTTCCTTACATAGAGTGCAGCTTTCAAGAAAAATTTCAATTTCTTCGTTTGTTGCATAATAACCACTGTCTTTGATATGATTAAACATTTTTGATTTTCTTAAATGCTTTCCTTC
TTTAATATGAAAATTATCGAATTCTAATTCATTAAGAACAATAAGCTCCCCTAATTTAAAAAATTAGTTAAAATAAATTAAAATGAACATGTATAAAGATGGATTTTACCATTTTTTGAAATTCTAA
ATAACTTTTCTTCATCTCCAATCTTTTTGACTGAAAAACGATTTTTAATTGAAGTTATTGTTCTGTGAGTGTTTTGAATCGCCCATTTCTCTAAATCAGTTTGAGATAGTGTTTTATAATCTGAATT
GTTATACACAACTTTTGCTCTATTAACCAAATATTTAAAGATTTCATCATCAACTGAATATTTTGACTTTACGATTCTTGTCCAAAAAACAATTTCTACTACTATCATTTTTTATTTATAAAATAAT
TTAAATACAAAAATGAATTTTTTTTTTTTTAAAAAAAAAAAAATTTGAAAAAAAAAAAAAAAAAATTTTAAAAAAAAAAAAAAAAAAAAAAAAAAAAAAAAAATCAAATAAAAAGTAAAAAATAAAA
ACCGAAAAACATTCATTGTAATTTCAAATGTCGAGGCCGGCAGAGGCGGTTTGCGTATTGGGCGCTCTTCCGCTTCCTCGCTCACTGACTCGCTGCGCTCGGTCGTTCGGCTGCGGCGAGCGGTATC
AGCTCACTCAAAGGCGGTAATACGGTTATCCACAGAATCAGGGGATAACGCAGGAAAGAACATGTGAGCAAAAGGCCAGCAAAAGGCCAGGAACCGTAAAAAGGCCGCGTTGCTGGCGTTTTTCCAT
AGGCTCCGCCCCCCTGACGAGCATCACAAAAATCGACGCTCAAGTCAGAGGTGGCGAAACCCGACAGGACTATAAAGATACCAGGCGTTTCCCCCTGGAAGCTCCCTCGTGCGCTCTCCTGTTCCGA
CCCTGCCGCTTACCGGATACCTGTCCGCCTTTCTCCCTTCGGGAAGCGTGGCGCTTTCTCATAGCTCACGCTGTAGGTATCTCAGTTCGGTGTAGGTCGTTCGCTCCAAGCTGGGCTGTGTGCACGA
ACCCCCCGTTCAGCCCGACCGCTGCGCCTTATCCGGTAACTATCGTCTTGAGTCCAACCCGGTAAGACACGACTTATCGCCACTGGCAGCAGCCACTGGTAACAGGATTAGCAGAGCGAGGTATGTA
GGCGGTGCTACAGAGTTCTTGAAGTGGTGGCCTAACTACGGCTACACTAGAAGGACAGTATTTGGTATCTGCGCTCTGCTGAAGCCAGTTACCTTCGGAAAAAGAGTTGGTAGCTCTTGATCCGGCA
AACAAACCACCGCTGGTAGCGGTGGTTTTTTTGTTTGCAAGCAGCAGATTACGCGCAGAAAAAAAGGATCTCAAGAAGATCCTTTGATCTTTTCTACGGGGTCTGACGCTCAGTGGAACGAAAACTC
ACGTTAAGGGATTTTGGTCATGAGATTATCAAAAAGGATCTTCACCTAGATCCTTTTAAATTAAAAATGAAGTTTTAAATCAATCTAAAGTATATATGAGTAAACTTGGTCTGACAGTTACCAATGC
TTAATCAGTGAGGCACCTATCTCAGCGATCTGTCTATTTCGTTCATCCATAGTTGCCTGACTCCCCGTCGTGTAGATAACTACGATACGGGAGGGCTTACCATCTGGCCCCAGTGCTGCAATGATAC
CGCGAGACCCACGCTCACCGGCTCCAGATTTATCAGCAATAAACCAGCCAGCCGGAAGGGCCGAGCGCAGAAGTGGTCCTGCAACTTTATCCGCCTCCATCCAGTCTATTAATTGTTGCCGGGAAGC
TAGAGTAAGTAGTTCGCCAGTTAATAGTTTGCGCAACGTTGTTGCCATTGCTACAGGCATCGTGGTGTCACGCTCGTCGTTTGGTATGGCTTCATTCAGCTCCGGTTCCCAACGATCAAGGCGAGTT
ACATGATCCCCCATGTTGTGCAAAAAAGCGGTTAGCTCCTTCGGTCCTCCGATCGTTGTCAGAAGTAAGTTGGCCGCAGTGTTATCACTCATGGTTATGGCAGCACTGCATAATTCTCTTACTGTCA
TGCCATCCGTAAGATGCTTTTCTGTGACTGGTGAGTACTCAACCAAGTCATTCTGAGAATAGTGTATGCGGCGACCGAGTTGCTCTTGCCCGGCGTCAATACGGGATAATACCGCGCCACATAGCAG
AACTTTAAAAGTGCTCATCATTGGAAAACGTTCTTCGGGGCGAAAACTCTCAAGGATCTTACCGCTGTTGAGATCCAGTTCGATGTAACCCACTCGTGCACCCAACTGATCTTCAGCATCTTTTACT
TTCACCAGCGTTTCTGGGTGAGCAAAAACAGGAAGGCAAAATGCCGCAAAAAAGGGAATAAGGGCGACACGGAAATGTTGAATACTCATACTCTTCCTTTTTCAATATTATTGAAGCATTTATCAGG
GTTATTGTCTCATGAGCGGATACATATTTGAATGTATTTAGAAAAATAAACAAATAGGGGTTCCGCGCACATTTCCCCGAAAAGTGCCACCTGACGCGCCCTGTAGCG
```

Supplementary Figure 1 (*continued*)

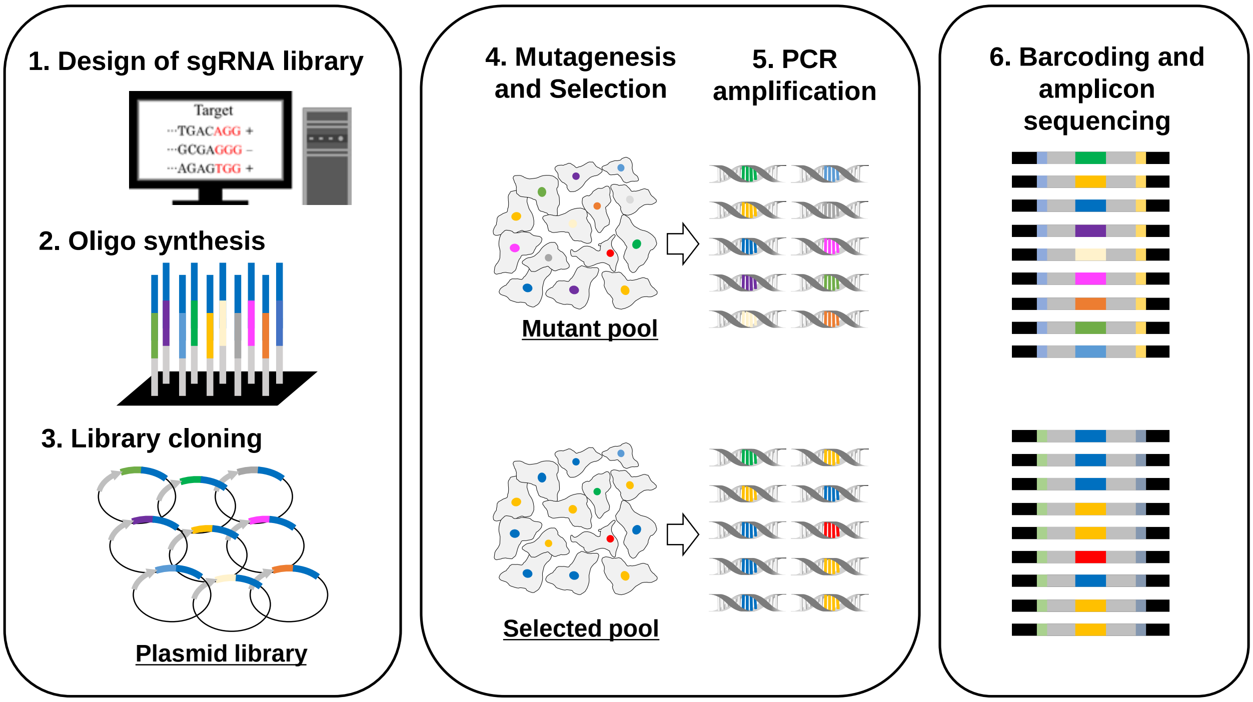

Supplementary Figure 2 General workflow of CRISPR screening.
1) In silico design of target sequences; **2)** Oligo synthesis; **3)** Cloning of plasmid sgRNA library; **4)** mutagenesis of genes in target cells and selection of mutants; **5)** Isolation of nucleic acids and PCR amplification; **6)** Deep sequencing of barcoded sgRNAs.

a

```
tra1          CATTTATTTTACCATTCCTTTATCAACGTTCATTAAATAATCCATCA
cl.1          CATTTATTTTACCATTTCTTTATCAACGTTCATTAAATAATCCATCA (+/-3)
cl.2          CATTTATTTTACCATT---TTATCAACGTTCATTAAATAATCCATCA (-3)
cl.3          CATTTATTTTACCATTCATTTATCAACGTTCATTAAATAATCCATCA (+/-3)
cl.4          CATTTATTTTACCATT---TTATCAACGTTCATTAAATAATCCATCA (-3)

atr1          TTCAAATTGATACCTTTTCTCTCACATAAATCATCAACAAACATTTG
cl.2          TTCAAATTGATACC-----TCTCACATAAATCATCAACAAACATTTG (-5)
cl.4          TTCAAATTGATACCTTT----TCACATAAATCATCAACAAACATTTG (-4)
cl.5          TTCAAATTGATACCTTTT--CTCACATAAATCATCAACAAACATTTG (-2)
cl.6          TTCAAATTGATACCTGATTTATGTGAGAGATACCTTTTTCTCACGAT (+23)

tor           CAGAATTCAGATGTTGATCCACAAATGATTGCATTGGCTTTGAAAAC
cl.1          CAGAATTCAGATGTTGATCCACAAT------CATTGGCTTTGAAAAC (+1/-7)
cl.2          CAGAATTCAGATGTTGAT------54bp insertion--------- (+60/-6)

DDB_G0278535  CCACTACCACCACCACCTCTGGTTGTGGTGGATTAAATGTACCAGAG
cl.1          CCACTACCACCACCACCT------GTGGTGGATTAAATGTACCAGAG (-6)
cl.2          CCACTACCACCACCACCT--GGTTGTGGTGGATTAAATGTACCAGAG (-2)
cl.3          CCACTACCACCACCACCTGTGGTTGTGGTGGATTAAATGTACCAGAG (+/-1)
cl.5          CCACTACCACCACCACCT--GGTTGTGGTGGATTAAATGTACCAGAG (-2)

roco5         ATTGATGGTATTCTATCACATCCATCAATTACCGTGGTCATTTTAAC
cl.1          ATTGATGGTATTCTATCACA-----------CCGTGGTCATTTTAAC (-11)
cl.2          ATTGATGGTATTCTATCACATCCATCAATTAAACCGTGGTCATTTTA (+2)
cl.3          ATTGATGGTATTCTATCACATCCATCAATTA-15bp insertion- (+15)
cl.4          ATTGATGGTATTCTATCACATCCATCAATTAATTAACACCGTGGTCA (+7)

gbpC          TAGTGAAATCGCCAAAGGTATGCAACATCTTCATTCTCATAATCCAC
cl.1          TAGTGAAATCGCCAAAG-TATGCAACATCTTCATTCTCATAATCCAC (-1)
```

b

```
Tra1          MLDQRTFQEVSTFILPFLYQRSLNNPSLLLIPQGFLSVTQMNPTGVQ
cl.1          MLDQRTFQEVSTFILPFLYQRSLNNPSLLLIPQGFLSVTQMNPTGVQ (L716L)
cl.2          MLDQRTFQEVSTFILPFIYQRSLNNPSLLLIPQGFLSVTQMNPTGVQ (L716I)
cl.3          MLDQRTFQEVSTFILPF-YQRSLNNPSLLLIPQGFLSVTQMNPTGVQ (-1aa)
cl.4          MLDQRTFQEVSTFILPF-YQRSLNNPSLLLIPQGFLSVTQMNPTGVQ (-1aa)

Tor           GGSIPQLGQNSDVDPQMIALALKTLGSFDFSKHNLLEFVRECVVNYL
cl.1          GGSIPQLGQNSDVDPQS--LALKTLGSFDFSKHNLLEFVRECVVNYL (M476S,-2aa)
cl.2          GGSIPQLGQNSDVDPQMTRSLRKVEKLKNSEPTLCTKLALKTLGSFD (+20aa/-2aa)
```

Supplementary Figure 3 Genomic mutations in recreated CRISPR mutants.
a) Sequencing results of recreated CRISPR mutants. The target gRNAs, PAM sequences and mutations are shown in blue, green and red, respectively. Numbers in parentheses indicate the number of modified nucleotides. Clones indicated by green letters were used for further analysis. **b)** Amino acid sequences of Tra1 and Tor. Mutated amino acids are shown in red underlines.

yakA	GGTTGGAACAGTTTATTGTGCATCAATTGATATGTGGTCATTG	
cl.P17	GGTTGGAACAGTTTATTGTG----------------GTCATTG	**(-16)**
pkaC	AAGGTCATGGTAAAGCGGTCGATTGGTGGGCACTTGGTATTCT	
cl.M19	AAGGTCATGGTAAAGCGGTCGATTGGTGGGCCCACTTGGTATT	**(+2)**
tor	GATCTCTGAGAGAGAATCACGTCTTCGTTTACAATGGTATCAA	
cl.K16	GATCTCTGAGAGAGAATCACGTCTTCGTTTAAACCAATGGTAT	**(+3)**
dhkG	AAGCTCAACGTGTTTTACATTTAGCATCTTGTATTGGTAATAG	
cl.O15	AAGCTCAACGTGTTTTACATTTAGCATCTTG-----GTAATAG	**(-5)**
snfA	GGTGCAATCTCTCAATTACCACCACATGAGATTATGGGTGAAA	
cl.J15	GGTGCAATCTCTCAATTACCACCACATGAGATATTATTATGGG	**(+5)**
dhkA	CTACTAATAGTCCAAGATTACTTGCCACTTCATTAAACAGTAC	
cl.N13	**-73bp insertion-**ATTACTTGCCACTTCATTAAACAGTAC	**(+73)**
mps1	GTGATTTGAAACCCGCAAATTTCGTCTCGGTTCAGGGTAGTTT	
cl.P13	GTGATTTGAAACCCGCAAATTTCGTCTCGGTTCAGGGTAGTTT	**(0)**
tsuA	AACCAATCCAACCAGTCCATCACCATCACCAAGAAGAAATAGT	
cl.L20	AACCAATCCAACCAGTC-ATCACCATCACCAAGAAGAAATAGT	**(-1)**
DDB_G0285517	GAAAATCTAAAAAGAGTGAACATGATGATATCAATGGAAATTC	
cl.H4	GAAAATCTAAAAAGAGTGAACATGATGAT--CAATGGAAATTC	**(-2)**
gcdH	GTCGCCTATGGTTTGATGGCAAACGCAGTAGAGAAGGTTGACA	
cl.C15	GTCGCCTATGGTTTGATGGCAAACGCAGTA-AGAAGGTTGACA	**(-1)**
gpaL	CATTGAAATTATTATTATTAGGTAGTGGTGAATGTGGTAAATC	
cl.F9	CATTGAAATTATTATTATTAGGTAGTGGTGAAT---------C	**(-9)**
DDB_G0293822	ATAAACTTTTAGTATTATCAGAGATACAATCATTAGGAAATAT	
cl.A24	ATAAACTTTTAGTATTATCAGAGATACA---ATTAGGAAATAT	**(-3)**
DDB_G0285613	AAAACAAGAGACCAAACCAAATAAAGATGAACTTGAAAGAGAC	
cl.D11	AAAACAAGAGACCAA-----ATAAAGATGAACTTGAAAGAGAC	**(-5)**
prosc	GTTAAAGAATTAATCTCAAGCTACAAGAATATAAAGGATAGAG	
cl.A9	GTTAAAGAATTAATCTCAAGCTACAAGAATA--AAGGATAGAG	**(-2)**
DDB_G0277541	TATCATAATGACCATTTCAGTGATATTAGTAACTCAATTCAAC	
cl.G18	TATCATAATGACCATTT--GTGATATTAGTAACTCAATTCAAC	**(-2)**

Supplementary Figure 4 Genome sequence analysis of identified gRNA regions.
Sequencing results of isolated CRISPR mutants were shown. The target gRNAs, PAM sequences and mutations are shown in blue, green and red, respectively. Numbers in parentheses indicate the number of modified nucleotides.

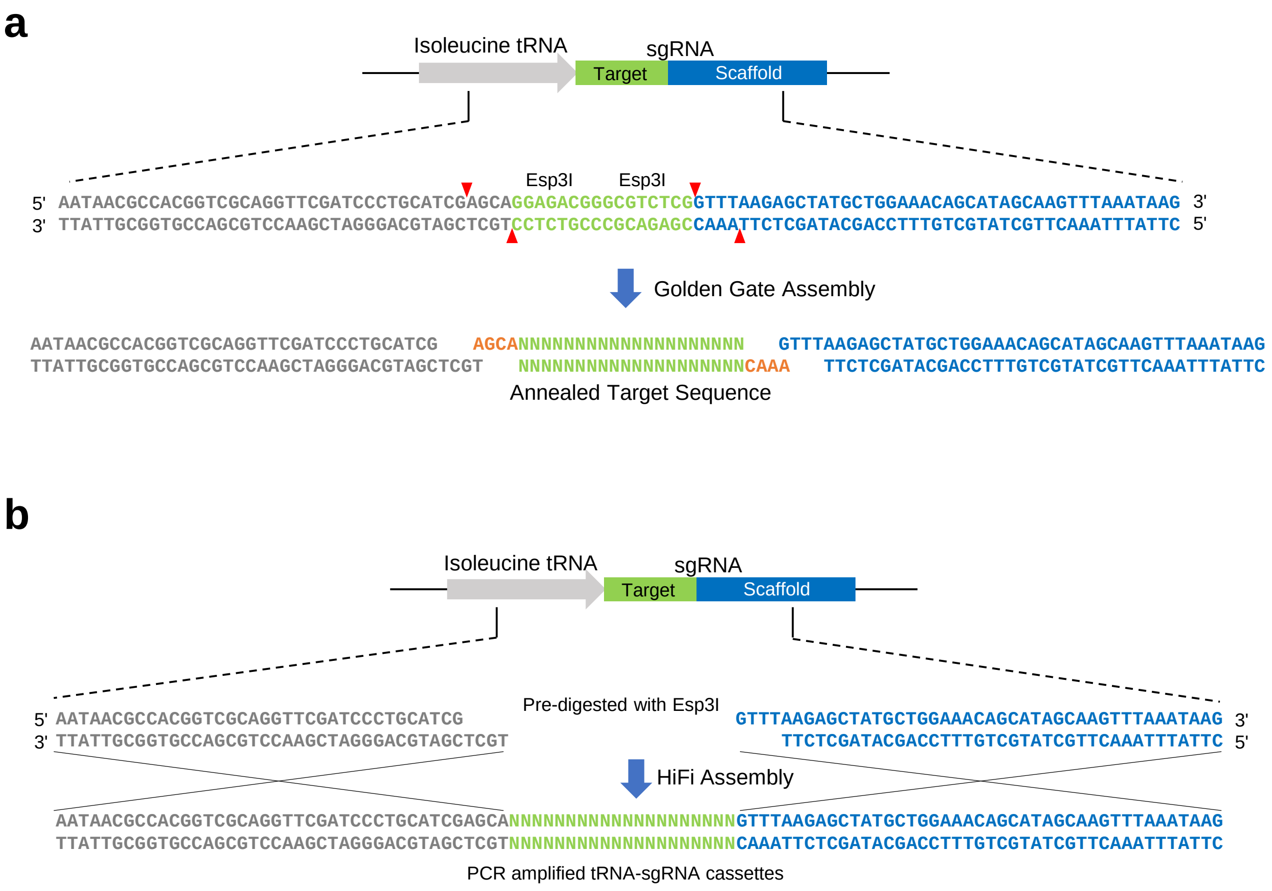

Supplementary Figure 5 Cloning of sgRNA containing target sequence.
a) Methods for cloning of individual target sequences into all-in-one CRISPR/Cas9 vector. Annealed target sequence was ligated to the tRNA-sgRNA junction via Golden Gate reaction. As type IIS restriction enzyme Esp3I site is non-palindromic, re-digestion is not possible. Orange letters show the overhangs for cloning. **b)** Schematic diagram of multi-sgRNA cloning. To clone multiple sgRNAs into all-in-one CRISPR/Cas9 vector, the pTM1709 or pTM1369 were digested with Esp3I. tRNA-sgRNA cassettes containing target sequences were amplified by PCR and the resulting fragments were assembled using a NEBuilder HiFi Assembly.

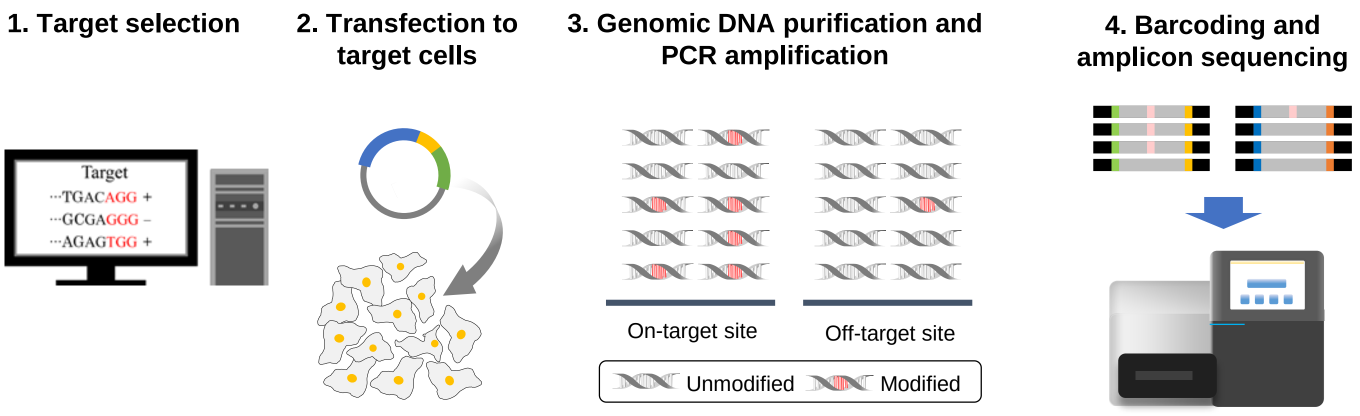

Supplementary Figure 6 Schematic workflow of the off-target detection method. **1)** Target sequences and off-target sites were predicted *in silico*. **2)** All-in-one CRISPR/Cas9 vectors were constructed and transfected into cells to induce specific mutations. **3)** gDNA was extracted from the cells and on-target and predicted off-target sites were amplified by PCR. **4)** The amplified DNA was barcoded and analysed by NGS.

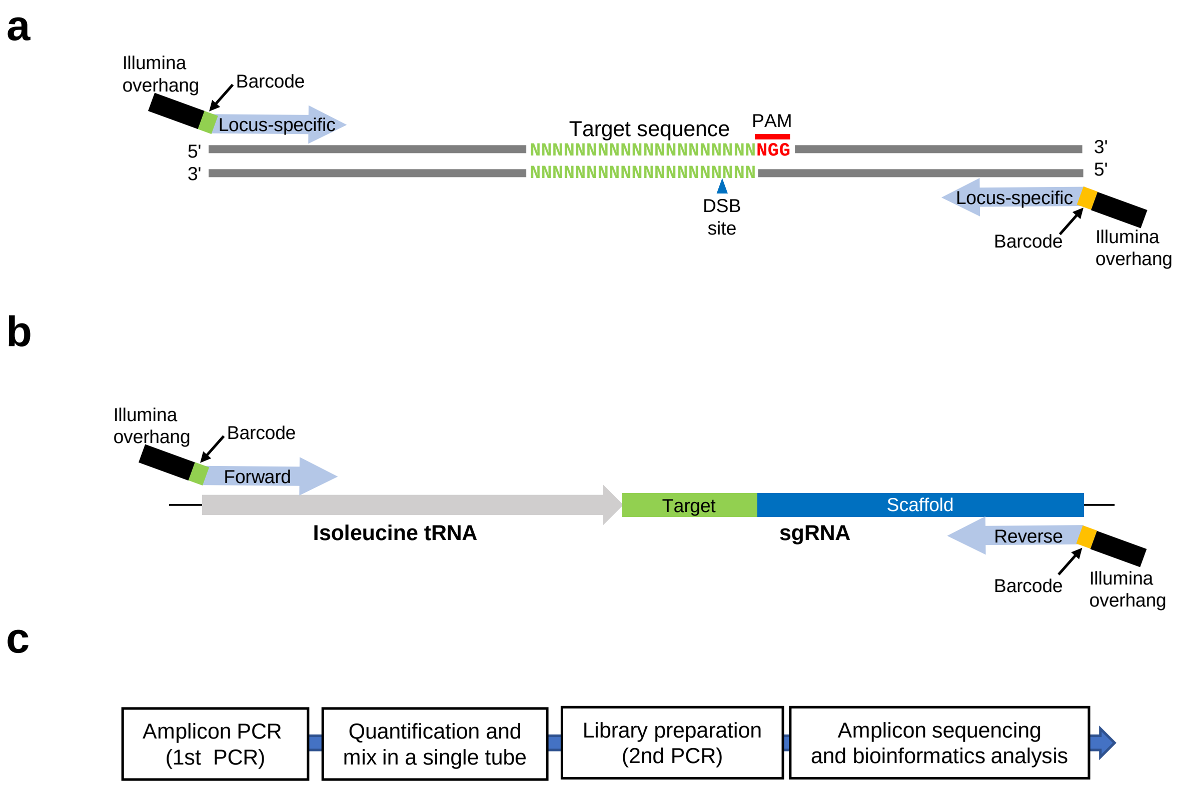

Supplementary Figure 7 Schematic workflow of the amplicon DNA library preparation with NGS index barcodes.

a) Amplicon PCR for off-target assay. In the first PCR, Illumina overhang (black) was added to the tail of the locus-specific primer. An index barcode sequence to distinguish the type of CRISPR vector used and the number of experimental trials was included between the illumine overhang and the locus-specific primer. **b)** Amplicon PCR to analyse sgRNA libraries and mutant pools. **(c)** Dual indexing strategies based on first and second PCR for amplicon sequencing.

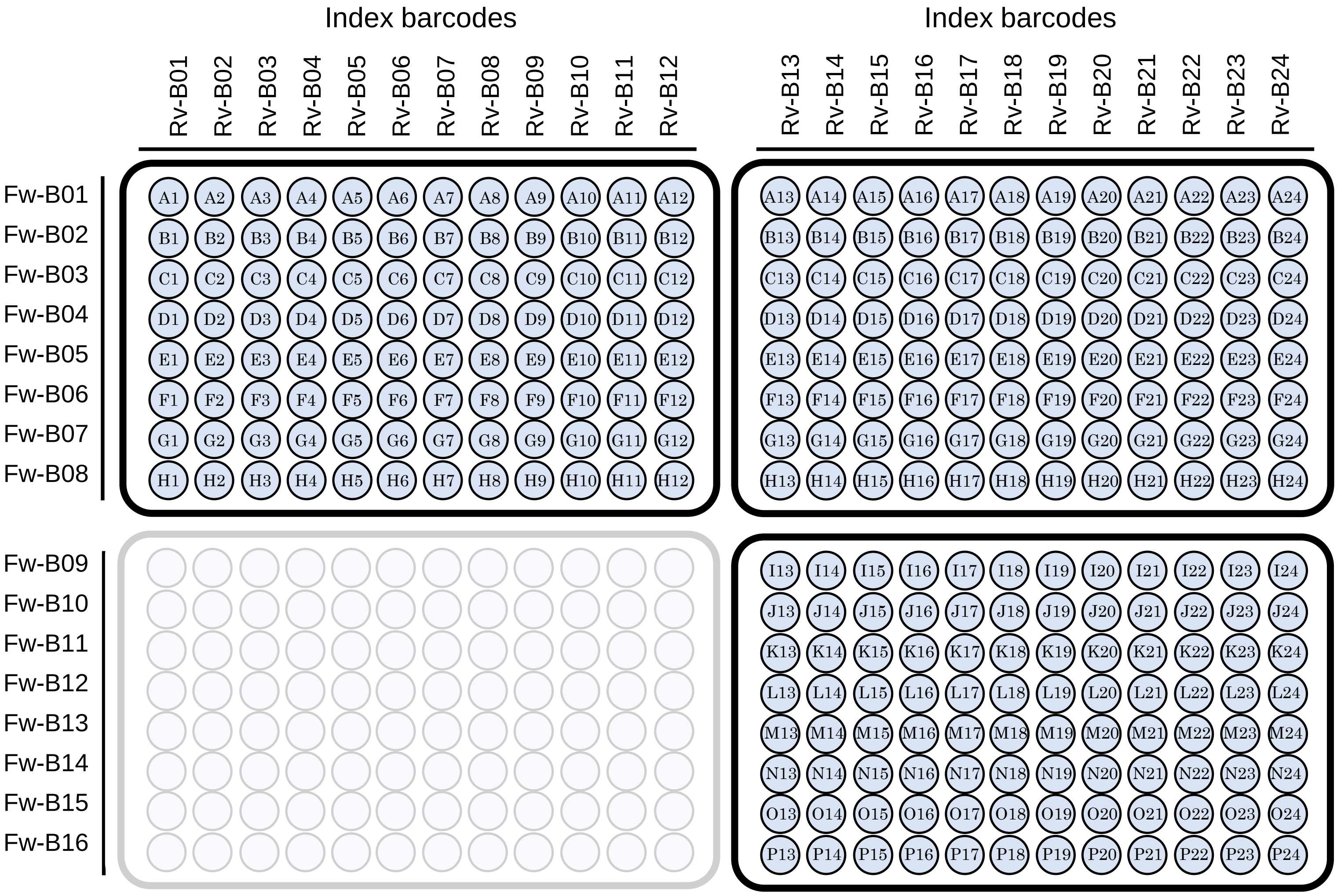

Supplementary Figure 8 Gridding of clonal mutants for amplicon sequencing.
Index barcodes in 16 forward and 24 reverse primers were used to distinguish PCR products derived from up to 384 clones. Pooled PCR products were used for addition of Illumina adaptors. Fw; forward primer, Rv; reverse primer.

Supplementary Table 1. GO analysis of the genes under-represented in genome-wide mutant pool.

GO ID	Term	Count	Fold Enrichment	Benjamini *p* value
GO:0005840	ribosome	14	13.03	2.42.E-09
GO:0003735	structural constituent of ribosome	15	11.09	2.73.E-09
GO:0006412	translation	16	8.78	1.85.E-08
GO:0022627	cytosolic small ribosomal subunit	7	23.23	1.90.E-05
GO:0031012	extracellular matrix	12	6.64	4.23.E-05
GO:1902600	hydrogen ion transmembrane transport	5	18.00	9.39.E-03
GO:0046961	proton-transporting ATPase activity, rotational mechanism	4	27.75	1.60.E-02
GO:0045335	phagocytic vesicle	12	3.37	1.66.E-02
GO:0022625	cytosolic large ribosomal subunit	5	10.82	2.14.E-02

Supplementary Table 3. GO analysis of the genes under-represented in growth selection.

# Term ID	Term description	Observed gene count	Background gene count	FDR
GO:0000075	Cell cycle checkpoint	2	31	0.033
GO:0051726	Regulation of cell cycle	3	148	0.023
GO:0048523	Negative regulation of cellular process	4	303	0.008
GO:0048519	Negative regulation of biological process	5	408	0.001
GO:0033554	Cellular response to stress	4	514	0.046
GO:0051716	Cellular response to stimulus	6	1078	0.007
GO:0050789	Regulation of biological process	8	1930	0.001
GO:0050794	Regulation of cellular process	7	1754	0.009

Supplementary Table 4. List of genes for which target gRNA sequences have been detected multiple times from independent mutants.

Gene	Count
yakA	31
pkaC	10
tor	4
dhkG	4
pats1	4
snfA	4
DDB_G0280131	3
dhkA	3
rckA	3
ndrD	3
DDB_G0277541	2
DDB_G0278521	2
DDB_G0279831	2
DDB_G0285517	2
DDB_G0285613	2
DDB_G0292350	2
dhkJ	2
dhkM	2
dnapkcs	2
dokA	2
fnkE	2
gcdh	2
gpaL	2
lvsG	2
mps1	2
mrkC	2
prosc	2
roco11	2
tsuA	2
vps15	2
DDB_G0283065	2
DDB_G0293822	2
mkcB	2

Supplementary Table 5. Recreated CRISPR-mediated mutants and observed phenotype.

No.	Gene	Target gRNA sequence (5′-3′)	Phenotype
1	*yakA*	ATTGTGCATCAATTGATATG	aggregation defect
2	*pkaC*	GCGGTCGATTGGTGGGCACT	aggregation defect
3	*tor*	AATCACGTCTTCGTTTACAA	aggregation defect
4	*dhkG*	TTACATTTAGCATCTTGTAT	delayed growth or development
5	*snfA*	ATTACCACCACATGAGATTA	aggregation defect
6	*dhkA*	AATGAAGTGGCAAGTAATCT	aberrant fruiting body
7	*mps1*	GCAAATTTCGTCTCGGTTCA	
8	*tsuA*	TCTTGGTGATGGTGATGGAC	small fruiting body
9	*mkcB*	CACCATATGCATTGGTGTCG	delayed growth or development
10	*DDB_G0285517*	AGTGAACATGATGATATCAA	
11	*gcdh*	GATGGCAAACGCAGTAGAGA	delayed growth or development
12	*gpaL*	TTATTAGGTAGTGGTGAATG	aggregation defect
13	*DDB_G0293822*	TTATCAGAGATACAATCATT	delayed growth or development
14	*DDB_G0285613*	AAGTTCATCTTTATTTGGTT	
15	*prosc*	CTCAAGCTACAAGAATATAA	
16	*DDB_G0277541*	AGTTACTAATATCACTGAAA	

Supplementary Table 6. List of oligonucleotides used to generate CRISPR/Cas9 all-in-one vectors for off-target assay.

On-target gene	Sequence (5'- to -3') Forward	Sequence (5'- to -3') Reverse
abcA6	agcaGCTTACAAACTGATATCATT	aaacAATGATATCAGTTTGTAAGC
grlJ	agcaAGAAACGTGCAATTACCAAT	aaacATTGGTAATTGCACGTTTCT
omt11	agcaGAAACAGTGGTTGATATTGG	aaacCCAATATCAACCACTGTTTC
DDB_G0285837	agcaTCCTCTTCAATTTGATCCCA	aaacTGGGATCAAATTGAAGAGGA

Supplementary Table 7. CRISPR/Cas9 all-in-one vectors for off-target assay.

Plasmid	On-target gene	Backbone vector	Cas9 type	Drug resistance
pTM1768	*abcA6*	pTM1709	Stable SpCas9	Blasticidin S
pTM1910	*grlJ*	pTM1709	Stable SpCas9	Blasticidin S
pTM1929	*omt11*	pTM1709	Stable SpCas9	Blasticidin S
pTM1950	*DDB_G0285837*	pTM1709	Stable SpCas9	Blasticidin S
pTM1954	*abcA6*	pTM1599	Transient SpCas9	G418
pTM1955	*grlJ*	pTM1599	Transient SpCas9	G418
pTM1956	*omt11*	pTM1599	Transient SpCas9	G418
pTM1957	*DDB_G0285837*	pTM1599	Transient SpCas9	G418
pTM1958	*abcA6*	pTM1668	Transient SpRY	G418
pTM1959	*grlJ*	pTM1668	Transient SpRY	G418
pTM1960	*omt11*	pTM1668	Transient SpRY	G418
pTM1961	*DDB_G0285837*	pTM1668	Transient SpRY	G418

Supplementary Table 8. List of primers used for off-target assay.

Gene	Forward / Reverse	Barcode index	Sequence (5'- to -3')
abcA5	Fw	index1	TCGTCGGCAGCGTCAGATGTGTATAAGAGACAG**CGTTACCAAC**AATCTCAATGTTAACTGGACT
abcA5	Fw	index2	TCGTCGGCAGCGTCAGATGTGTATAAGAGACAG**TGCGCAGTAC**AATCTCAATGTTAACTGGACT
abcA5	Fw	index3	TCGTCGGCAGCGTCAGATGTGTATAAGAGACAG**CCGTCAGTAC**AATCTCAATGTTAACTGGACT
abcA5	Fw	index4	TCGTCGGCAGCGTCAGATGTGTATAAGAGACAG**TGGTAACGAC**AATCTCAATGTTAACTGGACT
abcA5	Fw	index5	TCGTCGGCAGCGTCAGATGTGTATAAGAGACAG**ACTGCGCAAC**AATCTCAATGTTAACTGGACT
abcA5	Fw	index6	TCGTCGGCAGCGTCAGATGTGTATAAGAGACAG**ACTGACGGAC**AATCTCAATGTTAACTGGACT
abcA5	Rv	index1	GTCTCGTGGGCTCGGAGATGTGTATAAGAGACAG**TCGCCTTG**TCAATCTCATTTGCCATTTTCAA
abcA6	Fw	index1	TCGTCGGCAGCGTCAGATGTGTATAAGAGACAG**TAAGTAGAAC**GATTGGTATGTTGACAGGT
abcA6	Fw	index2	TCGTCGGCAGCGTCAGATGTGTATAAGAGACAG**TCGATAGCAC**GATTGGTATGTTGACAGGT
abcA6	Fw	index3	TCGTCGGCAGCGTCAGATGTGTATAAGAGACAG**ACGATCGAAC**GATTGGTATGTTGACAGGT
abcA6	Fw	index4	TCGTCGGCAGCGTCAGATGTGTATAAGAGACAG**TCTACTTAAC**GATTGGTATGTTGACAGGT
abcA6	Fw	index5	TCGTCGGCAGCGTCAGATGTGTATAAGAGACAG**GCTATCGAAC**GATTGGTATGTTGACAGGT
abcA6	Fw	index6	TCGTCGGCAGCGTCAGATGTGTATAAGAGACAG**TCGATCGTAC**GATTGGTATGTTGACAGGT
abcA6	Rv	index1	GTCTCGTGGGCTCGGAGATGTGTATAAGAGACAG**TCGCCTTG**TGTTGCCATTCTTTCAGCCT
DDB_G0285837	Fw	index1	TCGTCGGCAGCGTCAGATGTGTATAAGAGACAG**CAGGACGGCG**TTGCATCTGAACCAAAACC
DDB_G0285837	Fw	index2	TCGTCGGCAGCGTCAGATGTGTATAAGAGACAG**GATCGATCCG**TTGCATCTGAACCAAAACC
DDB_G0285837	Fw	index3	TCGTCGGCAGCGTCAGATGTGTATAAGAGACAG**GAAGAAGTCG**TTGCATCTGAACCAAAACC
DDB_G0285837	Fw	index4	TCGTCGGCAGCGTCAGATGTGTATAAGAGACAG**CCGTCCTGCG**TTGCATCTGAACCAAAACC
DDB_G0285837	Fw	index5	TCGTCGGCAGCGTCAGATGTGTATAAGAGACAG**GTCTGAGCCG**TTGCATCTGAACCAAAACC
DDB_G0285837	Fw	index6	TCGTCGGCAGCGTCAGATGTGTATAAGAGACAG**ACTTCTTCCG**TTGCATCTGAACCAAAACC
DDB_G0285837	Rv	index1	GTCTCGTGGGCTCGGAGATGTGTATAAGAGACAG**TCGCCTTG**TCATCATCATCCTGTTGTTGT
forE	Fw	index1	TCGTCGGCAGCGTCAGATGTGTATAAGAGACAG**GATGCACAGA**GCAAAGAAACCACCAGCA
forE	Fw	index2	TCGTCGGCAGCGTCAGATGTGTATAAGAGACAG**AGCTTGCCGA**GCAAAGAAACCACCAGCA
forE	Fw	index3	TCGTCGGCAGCGTCAGATGTGTATAAGAGACAG**GCAATCGAGA**GCAAAGAAACCACCAGCA
forE	Fw	index4	TCGTCGGCAGCGTCAGATGTGTATAAGAGACAG**TGTGCATCGA**GCAAAGAAACCACCAGCA
forE	Fw	index5	TCGTCGGCAGCGTCAGATGTGTATAAGAGACAG**GGCAAGCTGA**GCAAAGAAACCACCAGCA
forE	Fw	index6	TCGTCGGCAGCGTCAGATGTGTATAAGAGACAG**TCGATTGCGA**GCAAAGAAACCACCAGCA
forE	Rv	index1	GTCTCGTGGGCTCGGAGATGTGTATAAGAGACAG**TCGCCTTG**ACTTGAGTTGGCCCCTTTG
grlF	Fw	index1	TCGTCGGCAGCGTCAGATGTGTATAAGAGACAG**TGAGCATGCG**ATGGTAGTTGTCGTAGTAGAG
grlF	Fw	index2	TCGTCGGCAGCGTCAGATGTGTATAAGAGACAG**CAGATTGCCG**ATGGTAGTTGTCGTAGTAGAG
grlF	Fw	index3	TCGTCGGCAGCGTCAGATGTGTATAAGAGACAG**GTCATCAGCG**ATGGTAGTTGTCGTAGTAGAG
grlF	Fw	index4	TCGTCGGCAGCGTCAGATGTGTATAAGAGACAG**CATGCTCACG**ATGGTAGTTGTCGTAGTAGAG

grlF	Fw	index5	TCGTCGGCAGCGTCAGATGTGTATAAGAGACAG**GCAATCTGCG**ATGGTAGTTGTCGTAGTAGAG
grlF	Fw	index6	TCGTCGGCAGCGTCAGATGTGTATAAGAGACAG**TAGCCGTACG**ATGGTAGTTGTCGTAGTAGAG
grlF	Rv	index1	GTCTCGTGGGCTCGGAGATGTGTATAAGAGACAG**TCGCCTTG**TCAGCTACAATATCACCCAATGC
grlJ	Fw	index1	TCGTCGGCAGCGTCAGATGTGTATAAGAGACAG**ATCATGCTAT**TTGCACAATCACCAACCAA
grlJ	Fw	index2	TCGTCGGCAGCGTCAGATGTGTATAAGAGACAG**ATCGATAGAT**TTGCACAATCACCAACCAA
grlJ	Fw	index3	TCGTCGGCAGCGTCAGATGTGTATAAGAGACAG**TACGATCGAT**TTGCACAATCACCAACCAA
grlJ	Fw	index4	TCGTCGGCAGCGTCAGATGTGTATAAGAGACAG**AGCATGATAT**TTGCACAATCACCAACCAA
grlJ	Fw	index5	TCGTCGGCAGCGTCAGATGTGTATAAGAGACAG**CTATCGATAT**TTGCACAATCACCAACCAA
grlJ	Fw	index6	TCGTCGGCAGCGTCAGATGTGTATAAGAGACAG**CGATCGTAAT**TTGCACAATCACCAACCAA
grlJ	Rv	index1	GTCTCGTGGGCTCGGAGATGTGTATAAGAGACAG**TCGCCTTG**CAAACCTTGCCAAATAGCCAA
omt11	Fw	index1	TCGTCGGCAGCGTCAGATGTGTATAAGAGACAG**TCGCCTTGTG**GTGAAAATCAAGGACCTTCA
omt11	Fw	index2	TCGTCGGCAGCGTCAGATGTGTATAAGAGACAG**CGATTGCTTG**GTGAAAATCAAGGACCTTCA
omt11	Fw	index3	TCGTCGGCAGCGTCAGATGTGTATAAGAGACAG**CGATCGATTG**GTGAAAATCAAGGACCTTCA
omt11	Fw	index4	TCGTCGGCAGCGTCAGATGTGTATAAGAGACAG**CAAGGCGATG**GTGAAAATCAAGGACCTTCA
omt11	Fw	index5	TCGTCGGCAGCGTCAGATGTGTATAAGAGACAG**AGCAATCGTG**GTGAAAATCAAGGACCTTCA
omt11	Fw	index6	TCGTCGGCAGCGTCAGATGTGTATAAGAGACAG**ATCGATCGTG**GTGAAAATCAAGGACCTTCA
omt11	Rv	index1	GTCTCGTGGGCTCGGAGATGTGTATAAGAGACAG**TCGCCTTG**AGAAATCACCAGCAACATGTTT
omt9	Fw	index1	TCGTCGGCAGCGTCAGATGTGTATAAGAGACAG**ATTCTAGGTA**CACTGAAGCCGCCATATC
omt9	Fw	index2	TCGTCGGCAGCGTCAGATGTGTATAAGAGACAG**ATGACTGCTA**CACTGAAGCCGCCATATC
omt9	Fw	index3	TCGTCGGCAGCGTCAGATGTGTATAAGAGACAG**TCGCTAGATA**CACTGAAGCCGCCATATC
omt9	Fw	index4	TCGTCGGCAGCGTCAGATGTGTATAAGAGACAG**CCTAGAATTA**CACTGAAGCCGCCATATC
omt9	Fw	index5	TCGTCGGCAGCGTCAGATGTGTATAAGAGACAG**GCAGTCATTA**CACTGAAGCCGCCATATC
omt9	Fw	index6	TCGTCGGCAGCGTCAGATGTGTATAAGAGACAG**TCTAGCGATA**CACTGAAGCCGCCATATC
omt9	Rv	index1	GTCTCGTGGGCTCGGAGATGTGTATAAGAGACAG**TCGCCTTG**TGAGTTGGCCAATCATGAAGG

Index barcodes are shown in red letters.

Supplementary Table 9. List of primers for NGS analysis of kinase and genome-wide sgRNA plasmid libraries.

Name	Amplified plasmid	Sequence (5'- to -3')
sgRNAlibraryIndex1_Fw	pTM1376	TCGTCGGCAGCGTCAGATGTGTATAAGAGACAGTCGCCTTGCGATTAGCTCAGTCGGCAGAGCG
sgRNAlibraryIndex1_Rv	pTM1376	GTCTCGTGGGCTCGGAGATGTGTATAAGAGACAGATTCTAGGAGTTGATAACGGACTAGCCTTA
sgRNAlibraryIndex2_Fw	pTM1810	TCGTCGGCAGCGTCAGATGTGTATAAGAGACAGATAGCGTCCGATTAGCTCAGTCGGCAGAGCG
sgRNAlibraryIndex2_Rv	pTM1810	GTCTCGTGGGCTCGGAGATGTGTATAAGAGACAGCGTTACCAAGTTGATAACGGACTAGCCTTA

Index barcodes are shown in red letters.

Supplementary Table 10. List of primers for NGS analysis of kinase and genome-wide mutant pools.

Name	Mutant pool	Sequence (5'- to -3')
sgRNAlibraryIndex3_Fw	Kinase	TCGTCGGCAGCGTCAGATGTGTATAAGAGACAG**ATCATGCTTA**CGATTAGCTCAGTCGGCAGAGCG
sgRNAlibraryIndex4_Fw	Kinase	TCGTCGGCAGCGTCAGATGTGTATAAGAGACAG**GATGCACATCT**CGATTAGCTCAGTCGGCAGAGCG
sgRNAlibraryIndex5_Fw	Kinase	TCGTCGGCAGCGTCAGATGTGTATAAGAGACAG**CGATTGCTCGAC**CGATTAGCTCAGTCGGCAGAGCG
sgRNAlibraryIndex3_Rv	Kinase	GTCTCGTGGGCTCGGAGATGTGTATAAGAGACAG**GAAGAAGT**AGTTGATAACGGACTAGCCTTA
sgRNAlibraryIndex4_Rv	Kinase	GTCTCGTGGGCTCGGAGATGTGTATAAGAGACAG**ATGACTGC**AGTTGATAACGGACTAGCCTTA
sgRNAlibraryIndex5_Rv	Kinase	GTCTCGTGGGCTCGGAGATGTGTATAAGAGACAG**TGCACAGT**AGTTGATAACGGACTAGCCTTA
sgRNAlibraryIndex6_Fw	Genome-wide	TCGTCGGCAGCGTCAGATGTGTATAAGAGACAG**TCGATAGCAATTC**CGATTAGCTCAGTCGGCAGAGCG
sgRNAlibraryIndex7_Fw	Genome-wide	TCGTCGGCAGCGTCAGATGTGTATAAGAGACAG**ATCGATAGTTGCTT**CGATTAGCTCAGTCGGCAGAGCG
sgRNAlibraryIndex8_Fw	Genome-wide	TCGTCGGCAGCGTCAGATGTGTATAAGAGACAG**GATCGATCCAGTTAG**CGATTAGCTCAGTCGGCAGAGCG
sgRNAlibraryIndex6_Rv	Genome-wide	GTCTCGTGGGCTCGGAGATGTGTATAAGAGACAG**CGAATTGC**AGTTGATAACGGACTAGCCTTA
sgRNAlibraryIndex7_Rv	Genome-wide	GTCTCGTGGGCTCGGAGATGTGTATAAGAGACAG**GAGCACTT**AGTTGATAACGGACTAGCCTTA
sgRNAlibraryIndex8_Rv	Genome-wide	GTCTCGTGGGCTCGGAGATGTGTATAAGAGACAG**AGCTTGCC**AGTTGATAACGGACTAGCCTTA

Index barcodes are shown in red letters.

Supplementary Table 11. List of primer sets used for amplicon PCR of 384 samples.

Name	Sequence (5'- to -3')
Fw-B01	TCGTCGGCAGCGTCAGATGTGTATAAGAGACAG**TAAGTAGA**CGATTAGCTCAGTCGGCAGAGCG
Fw-B02	TCGTCGGCAGCGTCAGATGTGTATAAGAGACAG**ATCATGCTTA**CGATTAGCTCAGTCGGCAGAGCG
Fw-B03	TCGTCGGCAGCGTCAGATGTGTATAAGAGACAG**TCGCCTTG**CGATTAGCTCAGTCGGCAGAGCG
Fw-B04	TCGTCGGCAGCGTCAGATGTGTATAAGAGACAG**ATAGCGTC**CGATTAGCTCAGTCGGCAGAGCG
Fw-B05	TCGTCGGCAGCGTCAGATGTGTATAAGAGACAG**GTCTGATG**CGATTAGCTCAGTCGGCAGAGCG
Fw-B06	TCGTCGGCAGCGTCAGATGTGTATAAGAGACAG**TTACGCAC**CGATTAGCTCAGTCGGCAGAGCG
Fw-B07	TCGTCGGCAGCGTCAGATGTGTATAAGAGACAG**TTGAATAG**CGATTAGCTCAGTCGGCAGAGCG
Fw-B08	TCGTCGGCAGCGTCAGATGTGTATAAGAGACAG**AGCTTCAG**CGATTAGCTCAGTCGGCAGAGCG
Fw-B09	TCGTCGGCAGCGTCAGATGTGTATAAGAGACAG**GATGCACATCT**CGATTAGCTCAGTCGGCAGAGCG
Fw-B10	TCGTCGGCAGCGTCAGATGTGTATAAGAGACAG**CGATTGCTCGAC**CGATTAGCTCAGTCGGCAGAGCG
Fw-B11	TCGTCGGCAGCGTCAGATGTGTATAAGAGACAG**TCGATAGCAATTC**CGATTAGCTCAGTCGGCAGAGCG
Fw-B12	TCGTCGGCAGCGTCAGATGTGTATAAGAGACAG**ATCGATAGTTGCTT**CGATTAGCTCAGTCGGCAGAGCG
Fw-B13	TCGTCGGCAGCGTCAGATGTGTATAAGAGACAG**GATCGATCCAGTTAG**CGATTAGCTCAGTCGGCAGAGCG
Fw-B14	TCGTCGGCAGCGTCAGATGTGTATAAGAGACAG**CGATCGATTTGAGCCT**CGATTAGCTCAGTCGGCAGAGCG
Fw-B15	TCGTCGGCAGCGTCAGATGTGTATAAGAGACAG**ACGATCGATACACGATC**CGATTAGCTCAGTCGGCAGAGCG
Fw-B16	TCGTCGGCAGCGTCAGATGTGTATAAGAGACAG**TACGATCGATGGTCCAGA**CGATTAGCTCAGTCGGCAGAGCG
Rv-B01	GTCTCGTGGGCTCGGAGATGTGTATAAGAGACAG**TCGCCTTG**AGTTGATAACGGACTAGCCTTA
Rv-B02	GTCTCGTGGGCTCGGAGATGTGTATAAGAGACAG**GAAGAAGT**AGTTGATAACGGACTAGCCTTA
Rv-B03	GTCTCGTGGGCTCGGAGATGTGTATAAGAGACAG**ATTCTAGG**AGTTGATAACGGACTAGCCTTA
Rv-B04	GTCTCGTGGGCTCGGAGATGTGTATAAGAGACAG**CGTTACCA**AGTTGATAACGGACTAGCCTTA
Rv-B05	GTCTCGTGGGCTCGGAGATGTGTATAAGAGACAG**CAGGACGT**AGTTGATAACGGACTAGCCTTA
Rv-B06	GTCTCGTGGGCTCGGAGATGTGTATAAGAGACAG**ATGACTGC**AGTTGATAACGGACTAGCCTTA
Rv-B07	GTCTCGTGGGCTCGGAGATGTGTATAAGAGACAG**TGCACAGT**AGTTGATAACGGACTAGCCTTA
Rv-B08	GTCTCGTGGGCTCGGAGATGTGTATAAGAGACAG**CGAATTGC**AGTTGATAACGGACTAGCCTTA
Rv-B09	GTCTCGTGGGCTCGGAGATGTGTATAAGAGACAG**GAGCACTT**AGTTGATAACGGACTAGCCTTA
Rv-B10	GTCTCGTGGGCTCGGAGATGTGTATAAGAGACAG**AGCTTGCC**AGTTGATAACGGACTAGCCTTA
Rv-B11	GTCTCGTGGGCTCGGAGATGTGTATAAGAGACAG**CATTGCGA**AGTTGATAACGGACTAGCCTTA
Rv-B12	GTCTCGTGGGCTCGGAGATGTGTATAAGAGACAG**ACGTACGT**AGTTGATAACGGACTAGCCTTA
Rv-B13	GTCTCGTGGGCTCGGAGATGTGTATAAGAGACAG**CGGCTACA**AGTTGATAACGGACTAGCCTTA
Rv-B14	GTCTCGTGGGCTCGGAGATGTGTATAAGAGACAG**GTACATCT**AGTTGATAACGGACTAGCCTTA
Rv-B15	GTCTCGTGGGCTCGGAGATGTGTATAAGAGACAG**TCAGCTGC**AGTTGATAACGGACTAGCCTTA
Rv-B16	GTCTCGTGGGCTCGGAGATGTGTATAAGAGACAG**CCGTCAGT**AGTTGATAACGGACTAGCCTTA

Rv-B17	GTCTCGTGGGCTCGGAGATGTGTATAAGAGACAG**GACTGGAT**AGTTGATAACGGACTAGCCTTA
Rv-B18	GTCTCGTGGGCTCGGAGATGTGTATAAGAGACAG**TGTACCAG**AGTTGATAACGGACTAGCCTTA
Rv-B19	GTCTCGTGGGCTCGGAGATGTGTATAAGAGACAG**TACGGCTA**AGTTGATAACGGACTAGCCTTA
Rv-B20	GTCTCGTGGGCTCGGAGATGTGTATAAGAGACAG**GATCAGTC**AGTTGATAACGGACTAGCCTTA
Rv-B21	GTCTCGTGGGCTCGGAGATGTGTATAAGAGACAG**ATCGGCAT**AGTTGATAACGGACTAGCCTTA
Rv-B22	GTCTCGTGGGCTCGGAGATGTGTATAAGAGACAG**ACTCGGTA**AGTTGATAACGGACTAGCCTTA
Rv-B23	GTCTCGTGGGCTCGGAGATGTGTATAAGAGACAG**GTCATCAG**AGTTGATAACGGACTAGCCTTA
Rv-B24	GTCTCGTGGGCTCGGAGATGTGTATAAGAGACAG**TCGCTAGA**AGTTGATAACGGACTAGCCTTA

Index barcodes are shown in red letters.

Supplementary Data 1.

List of genes and gRNA target sequences used in genome-wide sgRNA library.

Supplementary Data 2.

List of gRNAs designed to increase the number of target genes.

Supplementary Data 3.

List of genes and gRNA target sequences used in kinase sgRNA library.

Supplementary Data 4.

Identified gRNA target sequences from individual developmental defective mutants.

Supplementary Table 2.

List of under- or over-represented genes by CRISPR screens for cell growth.
